# Revealing the co-existence of written and spoken language coding neural populations in the visual word form area

**DOI:** 10.1162/imag_a_00524

**Published:** 2025-03-31

**Authors:** Shuai Wang, Anne-Sophie Dubarry, Valérie Chanoine, Julien Sein, Jean-Luc Anton, Bruno Nazarian, Manuel R. Mercier, Agnès Trébuchon, Chotiga Pattamadilok

**Affiliations:** Aix Marseille University, CNRS, LPL, Aix-en-Provence, France; Aix Marseille University, Institute of Language, Communication and the Brain, Aix-en-Provence, France; Aix Marseille University, CNRS, CRPN, Marseille, France; Aix Marseille University, CNRS, Centre IRM-INT@ CERIMED, Institut de Neurosciences de la Timone, Marseille, France; Aix Marseille University, Inserm, INS, Inst Neurosci Syst, Marseille, France; APHM, Hôpital de la Timone, Service de Neurophysiologie Clinique, Marseille, France

**Keywords:** Repetition suppression, MVPA, fMRI, sEEG, neural representations, left-vOT

## Abstract

Reading relies on the ability to map written symbols with speech sounds. A specific part of the left ventral occipitotemporal cortex, known as the Visual Word Form Area (VWFA), plays a crucial role in this process. Through the automatization of the mapping ability, this area progressively becomes specialized in written word recognition. Yet, despite its key role in reading, the area also responds to speech. This observation raises questions about the actual nature of neural representations encoded in the VWFA and, therefore, the underlying mechanism of the cross-modal responses. Here, we addressed this issue by applying fine-grained analyses of within- and cross-modal repetition suppression effects (RSEs) and Multi-Voxel Pattern Analyses in fMRI and sEEG experiments. Convergent evidence across analysis methods and protocols showed significant RSEs and successful decoding in both within-modal visual and auditory conditions, suggesting that populations of neurons within the VWFA distinctively encode written and spoken language. This functional organization of neural populations enables the area to respond to both written and spoken inputs. The finding opens further discussions on how the human brain may be prepared and adapted for an acquisition of a complex ability such as reading.

## 1 Introduction

Reading is known to rely on a hierarchical organization of visual information processing: The ventral visual pathway distills information extracted from visual input in successive stages, from early visual cortices that process the physical aspect of the input to the occipitotemporal junction that processes information in a more abstract manner (Kravitz et al., 2013;Vinckier et al., 2007;Wandell et al., 2012;Yeatman & White, 2021). Within this process, the Visual Word Form Area (VWFA), located in the left ventral occipitotemporal cortex, is argued to play the central role in recognizing known scripts regardless of their physical characteristics, and lesions in this area generally lead to reading deficits (Bolger et al., 2005;Cohen & Dehaene, 2004;Cohen et al., 2000;Dehaene et al., 2001,^2010^;Gaillard et al., 2006;Nakamura et al., 2012;Szwed et al., 2014).

The anatomical location of the VWFA, interfacing the occipital and temporal cortex, as well as its connectivity pattern (Bouhali et al., 2014;Koyama et al., 2010;Saygin et al., 2016;Stevens et al., 2017;Wang et al., 2022;Yeatman et al., 2013) render the area ideal to support the exchanges between visual and non-visual information coming from the left-lateralized spoken language system and other parts of the brain. Several neuroimaging studies, indeed, showed that in addition to its key role in reading, the area also responds to non-visual language inputs. For instance, studies conducted on congenitally blind readers showed responses to Braille script in tactile modality (Reich et al., 2011) and to letter shapes coded by auditory soundscapes (Striem-Amit et al., 2012). Other studies conducted on the same population further showed that the area also responded to non-visual sensory inputs that could not be translated into spatial or shape patterns such as vowel sounds (Arnaud et al., 2013), spoken words (Dzięgiel-Fivet et al., 2021;Kim et al., 2017) or spoken sentences (Kim et al., 2017;Musz et al., 2022).

In the above examples, such functional reorganization which allows the visual cortex to respond to auditory inputs is mainly attributed to a deprivation of visual sensory input in blinds. Yet, at least in alphabetic languages, studies conducted on healthy participants also showed VWFA responses to speech in diverse tasks, ranging from those that required access to spelling knowledge, such as determining whether spoken words contain a target letter or share common rime spellings (English:Booth et al., 2002;Cao et al., 2010; German:Ludersdorfer et al., 2015,^2016^), to purely auditory processing tasks where an activation of orthographic representations is neither mandatory nor beneficial, such as spoken word recognition (Portuguese:Dehaene et al., 2010) and spoken sentence comprehension (French:Planton et al., 2019).

**Fig. 1.**
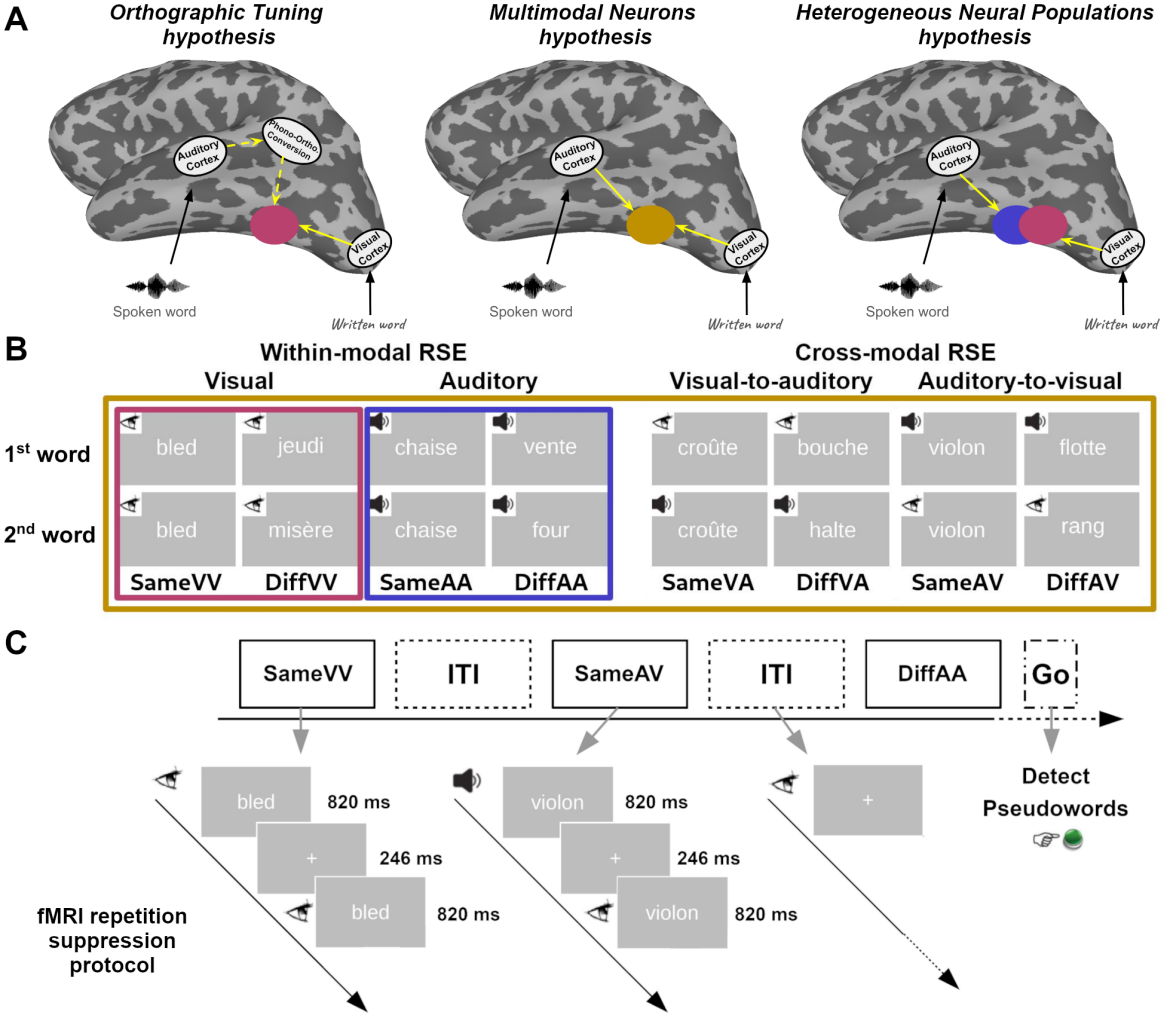
Hypotheses and repetition suppression protocols used in the fMRI and sEEG experiments. (A) Three hypotheses explaining the underlying mechanisms of VWFA responses to spoken inputs. (B) In the fMRI experiment, eight experimental conditions were used to assess the repetition suppression effects (RSEs). Within-modal visual RSE, within-modal auditory RSE, cross-modal visual-to-auditory RSE, and cross-modal auditory-to-visual RSE were assessed using the following contrasts, respectively:*DiffVV*vs.*SameVV*,*DiffAA*vs.*SameAA*,*DiffVA*vs.*SameVA*, and*DiffAV*vs.*SameAV*. The sEEG experiment had four experimental conditions, that is,*SameVV*,*SameAA*,*Same AV*, and*Same VA,*reflecting the same word presented twice in the same or different modality. Within-modal visual RSE, within-modal auditory RSE, cross-modal visual-to-auditory RSE, and cross-modal auditory-to-visual RSE were assessed by contrasting the first word versus the second word within the same-word pairs. (C) Flowchart illustrating the experiment procedure of the fMRI repetition suppression protocol.

Given the key role of the VWFA in reading, the observations that the area also responds to speech sounds raise questions about the property of neural populations within this specific area of the ventral visual pathway: Do these neural populations inherently respond to visual input only, and the reported cross-modal responses result from a conversion of non-visual inputs to their corresponding visual representations? Or, does the area also contain neural populations that respond to non-visual inputs? The answers to these questions, which should provide insightful information on how written and spoken information is processed and integrated in the area, vary across theoretical frameworks. So far, three main propositions have been put forward (Fig. 1A):*Orthographic Tuning hypothesis*,*Multimodal Neurons hypothesis*, and*Heterogeneous Neural Populations hypothesis*. As detailed below, although the three hypotheses could account for the VWFA responses to spoken input, they make different claims regarding the property of the neural responses in the area.

The*Orthographic Tuning hypothesis*(Fig. 1Aleft panel) proposed by Dehaene and colleagues (Cohen et al., 2004;Dehaene et al., 2005) described the VWFA as a unimodal visual region which, through reading acquisition, became progressively specialized in orthographic coding. This acquisition is assumed to make the area selective to known scripts. Although the authors supplemented a top-down mechanism that allows spoken inputs to activate orthographic coding neurons in the VWFA through the conversion of phonological to orthographic representations (Dehaene & Cohen, 2011;Dehaene et al., 2010), this hypothesis does not predict that the VWFA selectively responds to spoken inputs. An alternative view is proposed by the Interactive Account (Price & Devlin, 2003,^2011^) which considers the specific part of the ventral visual pathway as a convergence area that processes information from both visual regions and spoken language regions. Rather than (or in addition to) responding to orthographic representations generated from spoken inputs, the neural populations within this area could respond directly to spoken inputs. This account led to two hypotheses: The*Multimodal Neurons hypothesis*(Fig. 1Amiddle panel) assumes that the area contains multimodal neurons that respond to language inputs regardless of their modality (Price & Devlin, 2011). Alternatively, the*Heterogeneous Neural Populations hypothesis*(Fig. 1Aright panel) assumes that the area contains different types of unimodal neurons that distinctively encode written and spoken inputs (Price & Devlin, 2003).

In a previous study, Pattamadilok and colleagues (Pattamadilok et al., 2019) attempted to disentangle the three hypotheses, using a combination of noninvasive transcranial magnetic stimulation (TMS) and a neural adaptation protocol. Based on the assumption that the behavioral effects of TMS depend on the initial state of the neural populations being stimulated (state-dependent TMS effect,Cattaneo et al., 2009;Silvanto et al., 2008), the authors reported behavioral evidence (i.e., a facilitation of spoken vs. written word recognition) suggesting that neural populations within the VWFA, targeted by TMS, were able to adapt their responses to either written or spoken language input in a unimodal manner.

While the above behavioral evidence indirectly suggested that the VWFA might contain populations of neurons that distinctively encode either phonological or orthographic information, the present study used functional magnetic resonance imaging (fMRI: Experiment 1) and stereo-electroencephalography (sEEG: Experiment 2) to provide direct neural evidence on the nature of representations encoded in the area.

Experiment 1 involved two protocols. First, a repetition suppression protocol aimed to characterize the nature of neural representations encoded in the VWFA by examining within-modal (visual-to-visual; auditory-to-auditory) and cross-modal (visual-to-auditory; auditory-to-visual) repetition suppression effects (RSEs). As argued byBarron et al. (2016), repetition suppression provides a means to access the information content of neurons in the human brain thanks to an adaptation of neural responses to a stimulus. Neural adaptation is reflected in a reduction of brain activity in situations where a stimulus or an information feature is repeated relative to situations in which the preceding stimulus or information feature is different. The protocol has successfully been used to refine the functional resolution of the fMRI signal at the level of neural populations (see alsoGlezer et al., 2009;Grill-Spector et al., 2006).

Here, we applied this paradigm to examine the modality of language input encoded by neural populations along the ventral visual pathway, especially within the VWFA. If populations of neurons in this brain region encode a specific modality of language input, a repetition of a stimulus in that modality should lead to a reduction of BOLD response in these neural populations. The three hypotheses described above lead to three distinct predictions: 1) The*Orthographic Tuning hypothesis*predicts a reduction of VWFA activity only in the within-modal visual repetition condition, that is, where the same written words are repeated; 2) the*Multimodal Neurons hypothesis*predicts a similar reduction of VWFA activity in all repetition conditions, regardless of the modality of the inputs; and 3) finally, the*Heterogeneous Neural Populations hypothesis*predicts a reduction of VWFA activity in both within-modal visual and within-modal auditory repetition conditions, but not in the cross-modal conditions. Here, we examined the RSEs by comparing the brain response measured in a pair of same words to the brain response measured in a pair of different words presented in the same or different modality. The manipulation of word identity and modality resulted in eight conditions depicted inFigure 1B, and the predictions from the three hypotheses are listed inTable 1. In the second protocol, we conducted a lexical decision task on words and pseudowords presented in either visual or auditory modality. The aim was to complement the repetition suppression protocol by exploring multivariate within- and cross-modal decoding of language inputs according to their lexical category (word vs. pseudoword). In line with the predictions formulated above, the success of the different decoding scenarios (i.e., within-modal visual decoding, within-modal auditory decoding, and cross-modal decodings) would inform us about the nature of the representations encoded in the VWFA.

**Table 1.** Repetition suppression effects predicted by the three hypotheses.

	Repetition suppression effect (RSE)			
	Visual	Auditory	Visual-to-auditory	Auditory-to-visual
fMRI (comparisons between different-word pairs vs. same-word pairs)				
H _ORT_	DiffVV > SameVV			
H _MUL_	DiffVV > SameVV	DiffAA > SameAA	DiffVA > SameVA	DiffAV > SameAV
H _HET_	DiffVV > SameVV	DiffAA > SameAA		
sEEG (comparisons between the first vs. the second stimulus within the same-word pairs)				
	SameV _1_ V _2_	SameA _1_ A _2_	SameV _1_ A _2_	SameA _1_ V2
H _ORT_	V _1_ > V _2_			
H _MUL_	V _1_ > V _2_	A _1_ > A _2_	V _1_ > A _2_	A _1_ > V _2_
H _HET_	V _1_ > V _2_	A _1_ > A _2_		

Orthographic Tuning hypothesis (H_ORT_), Multimodal Neurons hypothesis (H_MUL_), Heterogeneous Neural Populations hypothesis (H_HET_).

Experiment 2 aimed to extend the finding observed in Experiment 1 by exploring the temporal dynamics of the neural populations that encode visual, auditory, or bimodal language input by recording intracranial stereotactic EEG (sEEG) in epileptic patients. To target these neural populations, we applied a similar repetition suppression protocol as in the fMRI experiment except that, thanks to the high temporal resolution offered by sEEG signals, we were able to record brain response to each stimulus and, therefore, to examine the RSEs within rather than across word pairs (see further details on the protocol in the Methods section,Fig. 1Bbottom andTable 1). Our analyses focused on high-frequency activity (HFA; 70–150 Hz), which is strongly correlated with the spiking activity of local neural populations (Buzsáki et al., 2012;Mercier et al., 2022). A significant reduction of HFA on the second compared to the first stimulus within the same-word pairs would indicate an RSE. A combination of these two measures (BOLD and HFA) allowed us to gain insight into the spatial distribution and temporal dynamics of within- and cross-modal RSEs.

## 2 Materials and Methods

### 2.1 Experiment 1: fMRI

The fMRI experiment aimed to delineate written and spoken language processing along the ventral pathway covering the VWFA and to disentangle the three hypotheses on the nature of representations processed by neural populations within this area. To this aim, we first identified the voxels within the VWFA that responded to written words in a visual localizer task, using a univariate analysis. Then, we further validated previous findings that the same VWFA voxels also responded to spoken inputs (Cao et al., 2010;Dehaene et al., 2010;Ludersdorfer et al., 2015,^2016^;Planton et al., 2019). Following these steps, we examined the property of the neural populations in the VWFA and along the ventral visual pathway through the occurrence of within- and cross-modal RSEs. Finally, to examine the neural representations in the VWFA from a complementary view, we applied multivariate pattern analysis (MVPA) to classify words from pseudowords both in within-modal (visual-to-visual; auditory-to-auditory) and cross-modal (visual-to-auditory; auditory-to-visual) decoding conditions.

#### 2.1.1 Participants

Twenty-two native French speakers participated in the study (age mean ± SD: 26.0 ± 4.3, 13 females). All participants were healthy, right-handed, with normal hearing and vision, and reported no neurological or language disorders. Written informed consent was obtained from all participants. The study was approved by the national ethics committee (CPP Sud-Méditerranée, no. ANSM, 2017-A03614-49).

#### 2.1.2 Stimuli

Word stimuli were mono- or disyllabic nouns selected from the French database LEXIQUE (http://www.lexique.org) with a minimal lexical frequency of 5 per million. In all tasks, when different subsets of words were used in different experimental conditions, they were matched on number of letters, number of syllables, number of phonemes, lexical frequency, OLD20, PLD20, and uniqueness point (seeSupplementary Table S1for detailed statistical properties of the stimuli and examples). All spoken inputs were recorded by a native French female speaker using an AKG C1000S microphone in an anechoic chamber (Centre d’Expérimentation sur la Parole, Laboratoire Parole et Langage, Aix Marseille University, CNRS), with a sampling rate of 48 kHz (32 bits).

##### 2.1.2.1 Visual localizer task

One hundred and ninety-two words were selected from the database, and 96 consonant strings were created by matching the number of letters with the words. All stimuli were presented in the visual modality.

##### 2.1.2.2 Auditory task

Ninety-six words were selected from the database and 96 pseudowords were generated by Wuggy (Keuleers & Brysbaert, 2010), using the same phonemes as in the words. Then, 96 scrambled stimuli were generated from the 96 spoken pseudowords by permuting Fourier components to remove phonological information while preserving acoustic information. All stimuli were presented in the auditory modality.

##### 2.1.2.3 Auditory task repetition suppression protocol

One hundred and forty-four words were selected from the database as NoGo trials and assigned to eight conditions as illustrated inFigure 1B. From this initial word pull, 72 words were generated in the visual modality and 72 words were generated in the auditory modalities. The aforementioned word properties were matched between the eight conditions (Kruskal-Wallis test; all*p*s > 0.49, seeSupplementary Table S1) and each word was used in only one condition, which allowed us to avoid cross-trial repetitions. Eight pseudowords (four in the visual modality and four in the auditory modality) were generated using Wuggy. They were used as Go trials.

##### 2.1.2.4 Lexical decision task

Sixty words were selected from the database and 60 pseudowords were generated by Wuggy (Keuleers & Brysbaert, 2010), using the same phonemes as in the words. All of the 120 stimuli were generated in both visual modality and auditory modality.

#### 2.1.3 Procedures

For each participant, we conducted a single scanning session in fMRI comprising four tasks: 1) a block-design visual localizer task to localize the voxels within the VWFA that responded to written words, 2) a block-design auditory task to examine whether the voxels identified in the visual localizer also responded to spoken inputs, 3) a repetition suppression protocol using an event-related pseudoword detection task in which pseudowords were randomly included among sequences of words which were presented in within- and cross-modal repetition suppression conditions (Fig. 1B), and 4) an event-related lexical decision task using both written and spoken words and pseudowords for MVPA.

##### 2.1.3.1 Visual localizer task

The visual localizer task was a block-design task presented in a single run that lasted 6.60 min. Words and consonant strings were presented in separate blocks of 11.8 s each. Each block contained 24 stimuli of the same condition. Each stimulus was displayed for 328 ms, followed by a 164 ms blank screen. Altogether, there were eight blocks of words and eight blocks of consonant strings, interleaved with 16 baseline blocks of “fixation” in which a cross remained on the screen for 11.8 s. No conditions were repeated twice in a row. During the task, participants were required to press a response button when they detected the target stimuli (######), which appeared randomly eight times between blocks. Each target stimulus lasted 328 ms and was followed by a fixation (jittered from ~1.2 s to 1.8 s) to allow responses. All stimuli were centrally presented on the screen in white font on a gray background.

##### 2.1.3.2 Auditory task

The auditory task was a block-design task presented in a single run that lasted 6.7 min. Spoken words, spoken pseudowords, and scrambled stimuli were presented in separate blocks of 12.1 s each. Each block contained 12 stimuli of the same condition. Each stimulus was presented for 804 ms, followed by a 201 ms silence. Altogether, there were eight blocks of spoken words, eight blocks of spoken pseudowords, eight blocks of scrambled stimuli, and eight silent-rest baseline blocks (which also lasted 12.1 s). All blocks were presented pseudo-randomly to avoid repetition of the same condition. During the task, participants were required to press a response button when they detected the target stimuli (beep sounds), which appeared randomly eight times between blocks. Each target stimulus lasted 335 ms, followed by a fixation (jittered from ~1.2 s to 1.8 s) to allow responses. All stimuli were presented through insert earphones.

##### 2.1.3.3 Repetition suppression protocol

The protocol was an event-related design with eight conditions based on the combinations of word identity (same vs. different) and modality (auditory/auditory, visual/visual, auditory/visual or visual/auditory), as illustrated inFigure 1B. The protocol allowed us to investigate four types of RSE: 1) Auditory RSE where all stimuli were presented in the auditory modality; 2) Visual RSE where all stimuli were presented in the visual modality; 3) Auditory-to-visual RSE where in both same-word and different-word pairs, the first stimulus was presented in the auditory modality and the second in the visual modality; and 4) Visual-to-auditory RSE where in both same-word and different-word pairs, the first stimulus was presented in the visual modality and the second in the auditory modality. As explained inTable 1, the four types of RSE were estimated by contrasting the brain activity measured during the processing of different-word pairs with the activity measured during the processing of same-word pairs. The RSE was expected to reflect a reduction of neural response to the same-word pairs compared to different-word pairs. The task was presented in two runs that lasted 8.2 min each. Each run contained six trials from each condition. The resulting 48 trials were presented pseudo-randomly to avoid repetitions of the same condition. Each trial lasted 1.89 s, including a pair of 820 ms words separated by a 246 ms interval. The inter-trial interval was jittered from 7.3 s to 9.6 s. During the task, the participants were not informed about the existence of word pairs and were required to press a button when they detected pseudowords (Go trials), which were randomly presented in either modality. The protocol is illustrated inFigure 1C.

##### 2.1.3.4 Lexical decision task

The lexical decision task was an event-related design with four conditions, that is, written words, written pseudowords, spoken words, and spoken pseudowords. The task was presented in five runs that lasted 3.8 min each. Each run contained 12 trials from each condition. The resulting 48 trials were pseudo-randomly presented, with a constraint that each stimulus was presented only once in each run in either visual or auditory modality. Each trial lasted 820 ms with an inter-trial interval jittered from 3.03 s to 4.2 s. During the task, the participants were required to determine whether a stimulus is a word or a pseudoword by pressing the corresponding button.

#### 2.1.4 Data acquisition

The experiment was conducted on a 3T Siemens Prisma Scanner (Siemens, Erlangen, Germany) at the Marseille MRI center (Centre IRM- INT@CERIMED, UMR7289 CNRS & AMU,http://irmf.int.univ-amu.fr/) using a 64-channel head coil. T1-weighted images were acquired using an MPRAGE sequence (voxel size = 1 × 1 × 1 mm^3^, data matrix = 256 × 240 × 192, TR/TI/TE = 2300/900/2.88 ms, flip angle = 9º, receiver BW=210 Hz/pix). Fieldmap images were obtained using Dual echo Gradient-echo acquisition (TR = 677 ms, TE1/TE2 = 4.92/7.38 ms, FOV = 210 × 210 mm^2^, voxel size = 2.2 × 2.2 × 2.5 mm^3^). Whole-brain functional images were collected during the auditory task using a gradient EPI sequence (TR = 1206 ms, TE = 30 ms, 54 slices with a thickness of 2.5 mm, FOV = 210 × 210 mm^2^, matrix = 84 × 84, flip angle = 65º, multiband factor = 3). Partial coverage functional images were collected during the other tasks to increase the spatial resolution of the ventral occipito-temporal cortex, using a gradient EPI sequence (TR = 1148 ms, TE = 32 ms, 42 slices with a thickness of 1.75 mm, FOV = 210 × 210 mm^2^, matrix = 114 × 114, flip angle = 66º, multiband factor = 3). Auditory stimuli were presented to the subject via the Sensimetrics S14 MR-compatible insert earphones with a Yamaha P-2075 power amplifier.

#### 2.1.5 Data pre-processing

Pre-processing was conducted by using fMRIPrep 20.2.0 (Esteban et al., 2019). For more details on the preprocessing pipeline, see fMRIPrep’s documentation (https://fmriprep.org/en/20.2.0/workflows.html) andSupplementary Text 1.

#### 2.1.6 Data analysis

Pre-processed functional data were scaled to percent of signal change and modeled using voxel-wise GLM for each task per participant. Motion contaminated volumes were identified and censored along with the prior volume if their FD > 0.5 mm. On average, 0.84% of the volumes were censored for the visual localizer task, 0.68% were censored for the auditory task, 0.61% were censored for the repetition suppression protocol, and 0.37% were censored for the lexical decision task. The six motion parameters, their temporal derivatives, and all their corresponding squared time series (i.e., 24 head motion regressors) were included in the GLMs to control for the impacts of head motion (Friston et al., 1996). In addition, the first six principal components of white matter and of CSF extracted by the aCompCor method (Behzadi et al., 2007) were used as nuisance regressors for the GLMs to reduce influence of physiological noise. The cosine-basis regressors estimated by fMRIPrep for high-pass filtering were also included in the GLMs as nuisance regressors.

##### 2.1.6.1 Regions of interest (ROIs) from the visual localizer task

Functional ROIs of the VWFA were extracted from T-maps contrasting*words*and*consonant strings*. T-maps were thresholded at both group and individual level. At the group level, the VWFA (ROI_GRP-VWFA_) was defined as ROI by identifying a single cluster of voxels through adjusting the significant threshold from a lenient*p*< 0.05 unc. to FDR q < 0.05 (equivalent*p*< 7.7e-4). At the individual level, the VWFA (ROI_IND-VWFA_) was defined as ROI by creating a sphere of 8 mm radius around the peak coordinates obtained from the individual contrast with*p*< 0.001. The mask of the ventral visual pathway (VVP mask) was defined by using the*words - fixation*contrast in the visual localizer with a lenient threshold,*p*< 0.01 uncorrected and then intersected with a pre-defined anatomical mask including the left inferior occipital, inferior temporal, fusiform, and lingual and parahippocampal gyri in the Automated anatomical labeling atlas (Rolls et al., 2020).

##### 2.1.6.2 Activation in the auditory task

The GLM for the auditory task included three regressors of interest -*spoken words*,*spoken pseudowords*, and*scrambled stimuli*. To reveal the response of VWFA to spoken inputs, three contrasts,*spoken pseudowords*–*scrambled stimuli*,*spoken words*–*scrambled stimuli*, and*spoken words*–*spoken pseudowords*, were estimated based on the GLMs.

##### 2.1.6.3 Repetition suppression effect

The GLM for the word repetition task included eight regressors of interest, that are*SameAA*,*DiffAA*,*SameVV*,*DiffVV*,*SameAV*,*DiffAV, SameVA*, and*DiffVA*. The average β value (percent of signal change) across all trials within each of the eight conditions was calculated for each participant. The auditory and visual RSEs were estimated by the contrasts of*DiffAA > SameAA*and*DiffVV > SameVV*, respectively. The cross-modal auditory-to-visual and visual-to-auditory RSEs were estimated by the contrast of*DiffAV > SameAV*and*DiffVA > SameVA*, respectively.

##### 2.1.6.4 Multi-voxel pattern analysis (MVPA)

Unlike repetition suppression, MVPA could assess collective responses in neural populations by showing a spatial activation pattern across multiple voxels (Hatfield et al., 2016;Norman et al., 2006;Oosterhof et al., 2013). As a complementary way to explore the neural representation within the VWFA, we carried out searchlight MVPA along the ventral visual pathway in a lexical decision task to classify words from pseudowords, based on within-modal (visual-to-visual or auditory-to-auditory) and cross-modal (visual-to-auditory or auditory-to-visual) information. Linear SVM and non-linear SVM classifiers were trained through leave-one-participant-out cross-validation in the following decoding conditions: 1) the classifier was trained and tested on written inputs: visual decoding; 2) it was trained and tested on spoken inputs: auditory decoding; 3) it was trained on written inputs and tested on spoken inputs: visual-to-auditory decoding; and 4) it was trained on spoken inputs and tested on written inputs: auditory-to-visual decoding. According to the*Orthographic Tuning hypothesis*, the VWFA was expected to show a successful (above-chance-level) classification performance in the within-modal visual condition. The*Heterogeneous Neural Populations hypothesis*predicted a successful decoding in both within-modal visual and auditory conditions. Finally, the*Multimodal Neurons hypothesis*predicted a successful decoding in all conditions.

In order to conduct MVPA, trial-wise estimates (i.e., β coefficients) were extracted for each condition in the lexical decision task by using the Least Squares — Separate (LSS) method (Mumford et al., 2012) which ran a GLM for each trial (3dLSS in AFNI). Trials with incorrect responses were excluded from the analysis, resulting in 9.7% trials being rejected on average. The MVPA was then conducted using Scikit-learn (Pedregosa et al., 2011) and Nilearn v0.10.1 (Abraham et al., 2014). Linear and non-linear SVM classifiers, Linear Discriminant Analysis (LDA), and Gradient Boosting Classifier (GBC) in Scikit-learn were trained with default parameters to classify words from pseudowords through leave-one-participant-out cross-validation. The searchlight with a sphere of 4 mm radius was applied within the ventral pathway mask defined by the visual localizer. The mask covered the VWFA functionally localized in our group of participants as well as those reported in the literature (Dehaene & Cohen, 2011;Dehaene et al., 2010;Jobard et al., 2003). The individual accuracy maps were entered in the group tests to compare to the chance-level accuracy (50%).

Unless stated otherwise, the voxel-level statistical comparisons in the present study were conducted by using AFNI’s*3dttest++*and*3dClustSim*with FWE cluster-based correction*p*< 0.05, voxelwise*p*< 0.005. The statistical maps were visualized with a highlighting approach (Taylor et al., 2023) to show sub-threshold results with decreasing opacity as the corresponding statistical significance decreases. The ROI-based statistical comparisons were conducted by using non-parametric pairwise permutation tests implemented in R and its*coin*(http://coin.r-forge.r-project.org/) and*rcompanion*(http://rcompanion.org/) packages and were FWE corrected per ROI. The effect size (Cohen’s d) and 95% CI were reported for significant ROI-based statistical comparisons (seeSupplementary Table S2for detailed effect sizes and 95% CI obtained for all comparisons).

### 2.2 Experiment 2: sEEG

To complement the fMRI experiment and explore the temporal dynamics of the VWFA response to spoken and written inputs, the sEEG data from four patients with electrodes implanted in the VWFA were analyzed. The modulation of high frequency activity (HFA) within- and cross-modal RSEs was measured as a proxy of population-level local spiking activity (Buzsáki et al., 2012;Mercier et al., 2022). To match the location of the areas within the ventral pathway across the fMRI and sEEG experiments, first, we pre-selected the electrodes that were located within a broad box mask covering all individual peaks found in the visual localizer task of the fMRI study (Supplementary Text 3,Supplementary Fig. S1A). Then, following the same rationale as in the fMRI experiment, we kept the electrodes showing significant HFA (70–150 Hz) to written words. The signals were then analyzed through a multi-patient permutation approach (Dubarry et al., 2017,^2022^).

#### 2.2.1 Participants

Eleven patients who underwent intracranial EEG monitoring for presurgical evaluation of epilepsy at the Hôpital de La Timone (Marseille, France) were recruited (age mean ± SD: 29.8 ± 12.2, six females). Four patients with electrodes located within the VWFA were included in the present study (age mean ± SD: 26.3 ± 5.7, two females; see sEEG Electrode Localization and Selection). None of the patients had previously undergone brain surgery. No seizures were observed within the 24 h preceding the experiment. Written informed consents were obtained from all patients. The study was approved by the Institutional Review Board of the French Institute of Health (IRB00003888).

#### 2.2.2 Stimuli

Critical word stimuli had the same characteristics as those described in Experiment 1. The spoken stimuli were also recorded under the same conditions. In this experiment, 200 words were selected and assigned to four same-word conditions of the repetition suppression protocol as illustrated inFigure 1B. Thus, from the initial pool of 200 words that had been selected, 50 words were in visual modality, 50 words were in auditory modality, and 100 words were in both modalities. The number of letters, number of syllables, number of phonemes, lexical frequency, OLD20, PLD20, and uniqueness point were matched between the four conditions (Kruskal-Wallis test; all*p*s > 0.40, seeSupplementary Table 1for detailed statistical properties of the stimuli and examples). In addition to the critical words, the material also contained other 400 fillers (50 written words, 50 written pseudowords, 50 consonant strings, 50 spoken words, 50 spoken pseudowords, 50 scrambled audio stimuli, 50 videos of lip-movements with speech sounds, and 50 videos of lip-movements without speech sounds). These stimuli were included here to reduce participants’ expectation on stimulus repetition and were analyzed in other studies.

#### 2.2.3 Procedures

The RSE protocol applied in this experiment was slightly different from the one used in the fMRI study: Each critical trial only contained the same word presented twice in a row either in the same modality or in different modality. Here, the time-course of neural activity was extracted for each word and the RSE was computed by contrasting the neural responses obtained on two consecutive stimuli.

The protocol contained four conditions of same-word pairs that were randomly presented among the fillers. The four conditions reflected the modality of the word that was repeated twice in a row (AA: both presentations were in the auditory modality, VV: both presentations were in the visual modality, AV: the first presentation was in the auditory modality and the second was in the visual modality, and VA: the first presentation was in the visual modality and the second was in the auditory modality), as illustrated inFigure 1B. Four types of RSE were estimated by contrasting the brain activity measured during the first and the second presentation of the word (Table 1). The task was presented in five blocks that lasted ~3.8 min each. Each block contained 10 critical trials (pair of words) from each condition. They were randomly presented among 80 fillers (10 per type of fillers) and 16 catch trials (eight ###### and eight beep sounds), with a constraint that no condition was presented twice in a row. The duration of the written inputs was 550 ms, while the duration of the spoken inputs depended on the file duration (ranged from 307 ms to 555 ms). The interval between two stimuli was jittered between 500 ms and 600 ms. During the task, the participants were not informed about the existence of word pairs and were requested to detect the catch trials by pressing the response button. Note that, to reduce fatigue and cognitive demands, the task applied on epileptic patients was simpler than the one applied in the fMRI study conducted on healthy participants.

#### 2.2.4 sEEG data acquisition

Intracerebral electrodes were implanted for clinical purposes, using 13 to 17 depth-electrodes, each containing 10 to 15 recording sites of 2 mm in length and separated by a 1.5 mm distance (0.8 mm in diameter; Alcis, Besançon, France). Patients were lying on their hospital bed with a laptop in front of them (approx. 80 cm). Data were recorded using a 256-channels Natus amplifier (Deltamed system) and sampled at 1000 Hz.

#### 2.2.5 sEEG electrode localization and selection

To precisely localize the channels, a procedure similar to the one used in the iELVis toolbox was applied (Groppe et al., 2017). First, we manually identified the location of each channel centroid on the post-implant CT scan using the Gardel software (Medina Villalon et al., 2018). Second, we performed volumetric segmentation and cortical reconstruction on the pre-implant MRI with the Freesurfer image analysis suite (http://surfer.nmr.mgh.harvard.edu/). Third, the post-implant CT scan was coregistered to the pre-implant MRI via a rigid affine transformation and the pre-implant MRI was registered to the MNI template (MNI 152 Linear), via a linear and a non-linear transformation from SPM12 methods, through the FieldTrip toolbox (Oostenveld et al., 2011). To identify sEEG contacts that are within the VWFA, a bounding box was created to cover this functional area by considering individual variability (Glezer & Riesenhuber, 2013). Specifically, the individual ROIs obtained in the fMRI visual localizer were used to define the boundaries of the bounding box, where X ∈ [-64.2, -25.4], Y ∈ [-72.1, -21.0] and Z ∈ [-35.8, -4.0] in MNI space. The sEEG electrode contacts located within the bounding box were further selected based on significant high-frequency activity to visual words to confirm the corresponding visual word processing.

#### 2.2.6 sEEG data analysis

The pre-processing of sEEG data was conducted using Brainstorm (Tadel et al., 2011; Version: 28-Jun-2022). For each patient, power spectral density (PSD) was estimated using Welch’s method with default setting in Brainstorm. The PSDs were visually inspected by two authors (SW and ASD) to identify outliers as noisy electrode contacts. None of the VWFA electrode contacts was rejected. Notch filters were applied at 50, 100, 150, 200, and 250 Hz. The signals were then high-pass filtered with 0.3 Hz using a Kaiser-window linear phase FIR filter with default setting of order 12376 to remove arbitrary DC offset and slow drift. The monopolar and bipolar data were visually inspected for marking bad segments by two authors (WS and ASD) who were blinded to trial labels. The signals were then segmented into epochs of -300 ms to 600 ms locked to stimulus onset. These epochs were imported in Multi-patient Intracerebral data Analysis (MIA) toolbox (Dubarry et al., 2022) for estimating high-frequency activity (HFA; 70–150 Hz with steps of 10 Hz) to conduct multi-patient permutation tests. All analyses were conducted using a bipolar montage with a seven cycles Morlet wavelet and a baseline from 300 ms to 10 ms before trial onset to exclude edge effects. The multi-patient permutation tests were conducted on individual electrode contacts for each type of RSE with 1000 iterations and the significant threshold*p*< .05 (Anders et al., 2019;Dubarry et al., 2017,^2022^Supplementary Text 2).

## 3 Results

### 3.1 Experiment 1: fMRI

#### 3.1.1 Identification of the regions-of-interest: the visual word form area

**Fig. 2.**
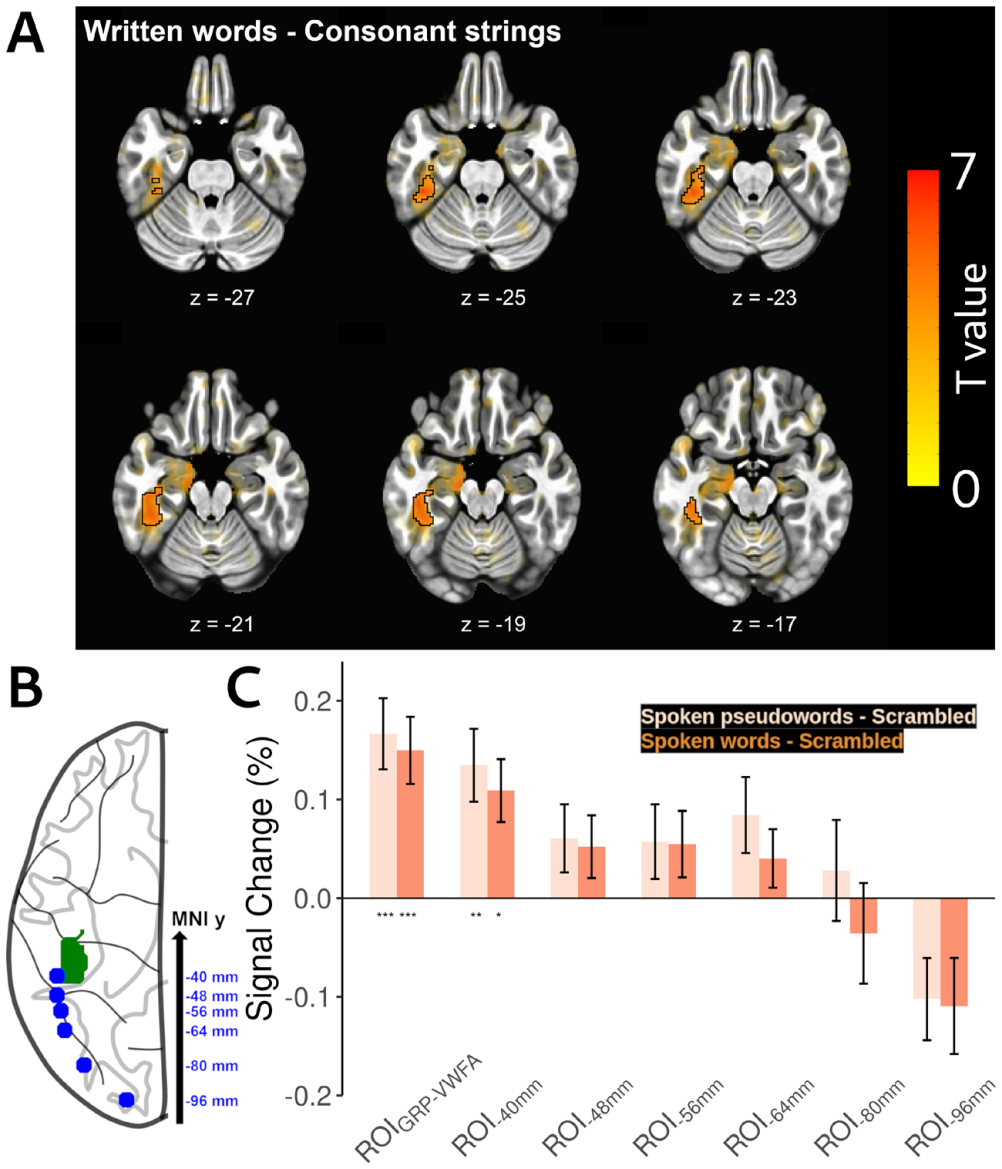
VWFA activation in response to written and spoken inputs. (A) Significant activation in the VWFA revealed by the*written words*–*consonant strings*contrast from the visual localizer (FDR q < 0.05; highlighted by black contour). Sub-threshold results were shown outside black contours and visible with decreasing opacity as the corresponding statistical significance decreases. (B) Axial view showing the locations of ROI_GRP-VWFA_(green patch) and the six literature-based ROIs along the ventral pathway (blue dots). (C) Both ROI_GRP-VWFA_and ROI_-40 mm_were significantly activated in the*spoken pseudowords*–*scrambled stimuli*(light orange bars) and*spoken words*–*scrambled stimuli*contrasts (dark orange bars). This activation induced by spoken inputs progressively increased from the posterior to the anterior portions of the visual pathway. Error bars represent standard errors. *p< 0.05; **p< 0.01; ***p< 0.005 (permutation tests with FWE correction for each ROI).

To functionally localize our region-of-interest, we first identified the voxels that responded to written inputs at the group level, using the*words*–*consonant strings*contrast from the visual localizer task. The voxel-wise comparison revealed a significant cluster (FDR q < 0.05; peak MNI -42, -36, -24;Fig. 2A), which was considered in the subsequent analyses as the region-of-interest, that is, VWFA (henceforth, ROI_GRP-VWFA_;Fig. 2B). In addition to the group-specific ROI identified in the visual localizer task, analyses were conducted on the literature-based ROIs to have a more global view of the neural response along the ventral visual pathway. To this end, we selected six ROIs fromVinckier et al. (2007)showing a gradient of selectivity from low-level visual features to written words. Those ROIs, located from the anterior to posterior parts of the ventral visual pathway, are referred to as ROI_-40 mm_, ROI_-48 mm_, ROI_-56 mm_, ROI_-64 mm_, ROI_-80 mm_and ROI_-96 mm_according to their y coordinates (Fig. 2B). Additionally, in the Supplementary Information (Supplementary Text 3 and Text 4,Supplementary Fig. S1), we presented the analyses conducted on individually defined ROIs (ROI_IND-VWFA_). These results confirmed those obtained in the ROI_GRP-VWFA_.

#### 3.1.2 Replication of the VWFA activation during spoken language processing

Following the ROI identification described above, we examined the VWFA activation in response to spoken inputs using the*spoken pseudowords*–*scrambled stimuli*,*spoken words*–*scrambled stimuli*, and*spoken words*–*spoken pseudowords*contrasts from the auditory task. The ROI-based analysis using permutation tests with FWE correction for each ROI (Fig. 2C) showed that the ROI_GRP-VWFA_and ROI_-40 mm_were significantly activated in both*spoken pseudowords*–*scrambled stimuli*contrast (*p*< 0.003, Cohen’s d: 1.29, 95% CI: 0.62, 1.95 for ROI_GRP-VWFA_;*p*< 0.011, Cohen’s d: 1.00, 95% CI: 0.35, 1.65 for ROI_-40 mm_) and*spoken words*–*scrambled stimuli*contrasts (*p*< 0.002, Cohen’s d: 1.31, 95% CI: 0.64, 1.98 for ROI_GRP-VWFA_;*p*< 0.008, Cohen’s d: 0.99, 95% CI: 0.34, 1.63 for ROI_-40 mm_. No significant difference was found in the*spoken words - spoken pseudowords*contrast (all*p*s > 0.058). A similar result was also obtained from the voxel-wise analysis on whole-brain (Supplementary Text 5,Supplementary Fig. S2). Therefore, we successfully replicated previous findings showing that VWFA voxels that respond to written inputs are also involved in the processing of spoken inputs (Cao et al., 2010;Dehaene et al., 2010;Ludersdorfer et al., 2015,^2016^;Planton et al., 2019). Interestingly, as shown inFigure 2C, such response increased progressively from posterior to anterior portions of the ventral visual pathway.

#### 3.1.3 Investigating the underlying mechanism(s) of VWFA responses to spoken inputs using within- and cross-modal repetition suppression effects

In the above analysis, we successfully replicated existing observations of the VWFA response to speech sounds. Here, we attempted to tease apart the three hypotheses on the underlying mechanism(s) of such activation by examining the within- and cross-modal RSEs in the ROIs defined above.

**Fig. 3.**
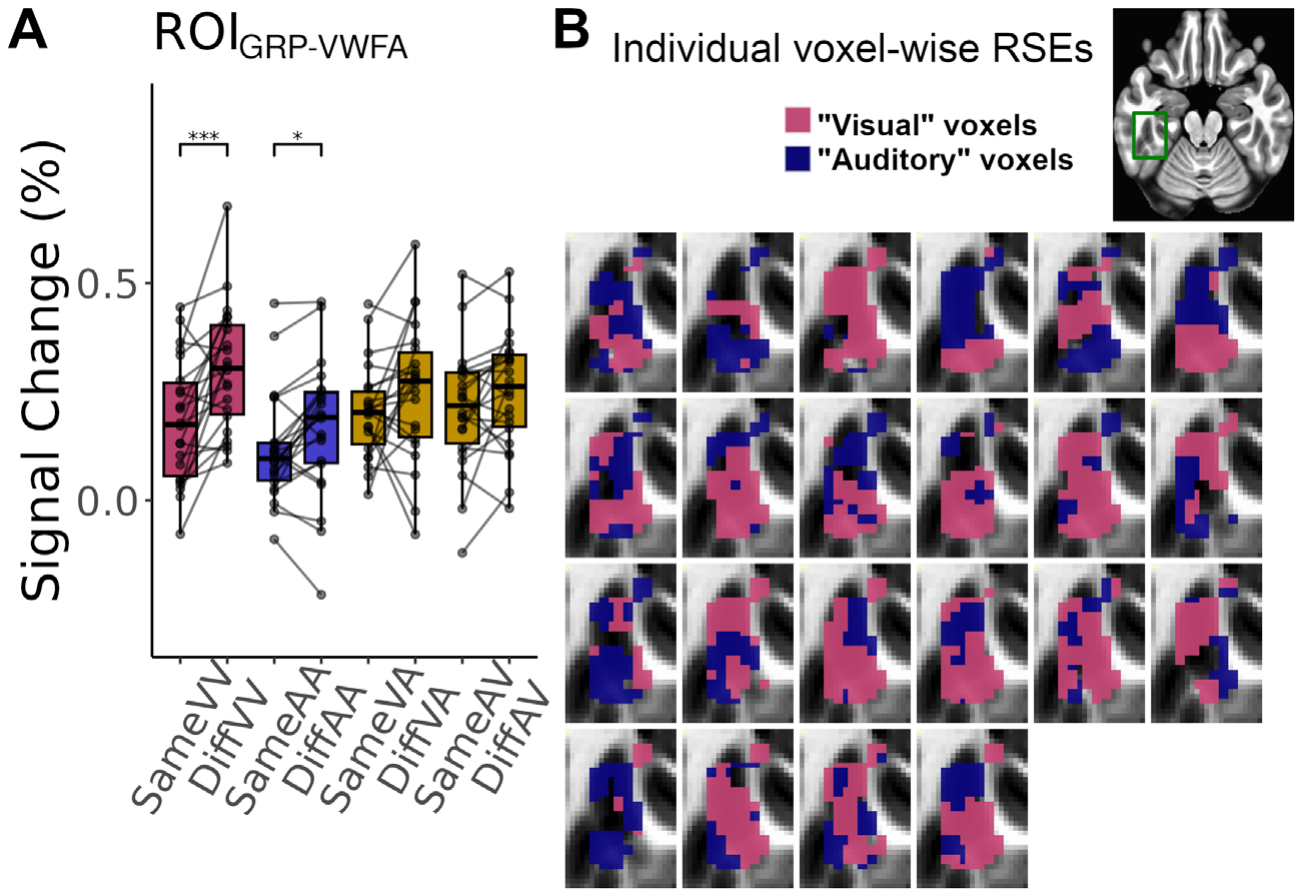
Repetition suppression effects in the VWFA. (A) Within-modal visual (pink bars) and within-modal auditory RSEs (purple bars) were significant in the ROI_GRP-VWFA_. No cross-modal RSEs (brown bars) were found. *p< 0.05; ***p< 0.005 (permutation tests with FWE correction). (B) Within the ROI_GRP-VWFA_, the winner-takes-all categorization of voxels showed a strong inter-individual variability of the distribution of the voxels with a preference for within-modal visual RSE (“visual” voxels) or within-modal auditory RSE (“auditory” voxels) (MNI y = -21). The spatial location of the depicted results is indicated by the green frame in the axial view in the top-right panel.

To test the directional hypotheses of RSE (Table 1), we conducted the ROI-based analyses using one-tailed permutation tests with FWE correction for each ROI. The result of ROI_GRP-VWFA_(Fig. 3A) showed significant within-modal RSEs in both visual (*DiffVV*>*SameVV*,*p*< 0.0033, Cohen’s d: 0.85, 95% CI: 0.21, 1.48) and auditory modalities (*DiffAA*>*SameAA*,*p*< 0.042, Cohen’s d: 0.33, 95% CI -0.28, 0.94). However, no significant cross-modal RSE was observed (all*p*s > 0.065). Following this observation, we further explored, at the voxel level, the distribution of clusters of voxels that showed a preference for either within-modal auditory RSE or within-modal visual RSE in the ROI_GRP-VWFA_by using the winner-takes-all approach (Mattioni et al., 2020;Van Den Hurk et al., 2017). Precisely, for each participant, the β-values were computed for within-modal visual RSE (*DiffVV*-*SameVV*) and within-modal auditory RSE (*DiffAA*-*SameAA*) within each voxel.Figure 3Billustrates the clusters of voxels that showed higher β-values for within-modal visual RSE (in pink) and for within-modal auditory RSE (in purple). The analysis showed a high inter-individual variability in voxel-wise distribution of the two types of within-modal RSE.

**Fig. 4.**
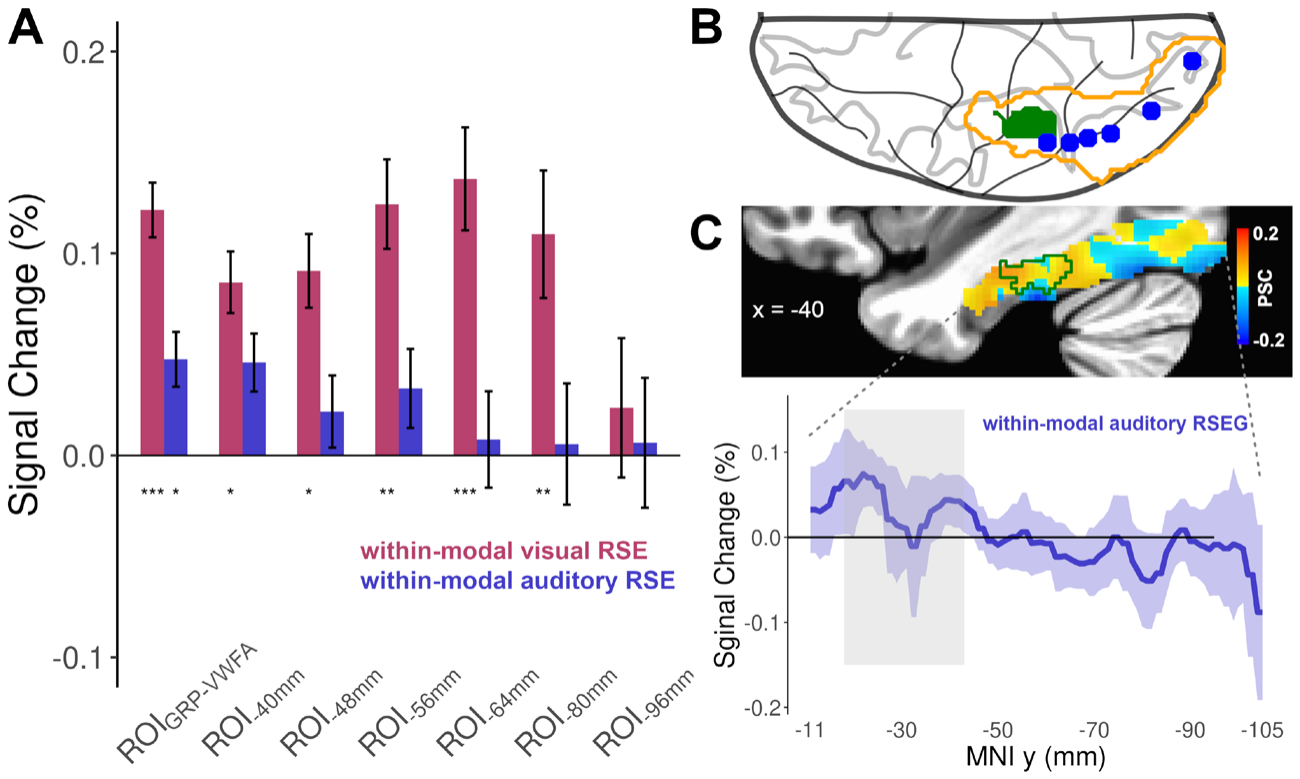
Repetition suppression effects in the ventral visual pathway. (A) The ROI_GRP-VWFA_showed significant within-modal visual and within-modal auditory RSEs (permutation tests with FWE correction for each ROI). The literature-based ROIs from -80 mm to -40 mm showed significant within-modal visual RSEs, with a trend of a gradual increase of within-modal auditory RSE from the posterior to anterior portions of the ventral visual pathway. *p< 0.05; **p< 0.01; ***p< 0.005 (permutation tests with FWE correction). (B) Axial view showing the location of the ventral visual pathway mask (orange contour) that contains ROI_GRP-VWFA_(green patch) and the six literature-based ROIs (blue dots). (C) The brain map illustrates the group-averaged within-modal auditory RSE along the ventral visual pathway (MNI x = -40). The color scale indicates the percent signal change (yellow-to-red indicates positive within-modal auditory RSE). The green contour in the brain map indicates ROI_GRP-VWFA_. The bottom panel shows the averaged β-values of the within-modal auditory RSE extracted from each slice along the y-axis of the ventral visual pathway mask. Blue ribbon represents standard errors. Gray rectangle indicates the y-axis range of ROI_GRP-VWFA_.

The analyses conducted on the six literature-based ROIs along the ventral visual pathway, using permutation tests with FWE correction for each ROI, showed significant within-modal visual RSEs from ROI_-80 mm_to ROI_-40 mm_(*DiffVV*>*SameVV*:*p*s < 0.012, Cohen’s d ≥ 0.35, 95% CIs all within the range of -0.26, 0.97). Although no significant within-modal auditory RSE was found in any ROIs (all*p*s > 0.097), their activation profiles showed a trend of an increased RSE for spoken words from the posterior to anterior portions of the pathway (Fig. 4A). To further illustrate this trend, the averaged β-values were extracted for the within-modal auditory RSE (*DiffAA - SameAA*) from each slice along the y-axis of a mask of the ventral visual pathway (VVP maskFig. 4B). The result confirmed the gradual increase of RSE for spoken words from posterior to anterior regions (Fig. 4C), indicating that the VWFA might act as a transition area where populations of neurons that respond to spoken or to written language input intermingled.

It is noteworthy that the absence of the significant cross-modal RSE in the analyses conducted on our region-of-interest does not call into question the validity of this type of RSE. Indeed, the same analysis conducted on a multimodal language area in left posterior superior temporal sulcus (pSTS) taken from a meta-analysis study on audiovisual integration (Erickson et al., 2014) showed significant RSEs in the four conditions as expected in a multimodal language area. Also as a sanity check, we examined the RSEs in the primary auditory cortex: The area only showed the expected significant within-modal auditory RSEs. These findings which confirmed the validity of the present repetition suppression protocol are reported in theSupplementary Text 6,Supplementary Figure S3.

#### 3.1.4 Investigating the underlying mechanism(s) of the VWFA responses to spoken inputs using within-modal and cross-modal MVPA decoding of stimulus lexicality

The above results suggest the existence of spoken language coding neural populations in the VWFA by showing a suppression of local neural responses to repeated spoken words. We further assessed the collective responses of neural populations in the VWFA using searchlight MVPA.

**Fig. 5.**
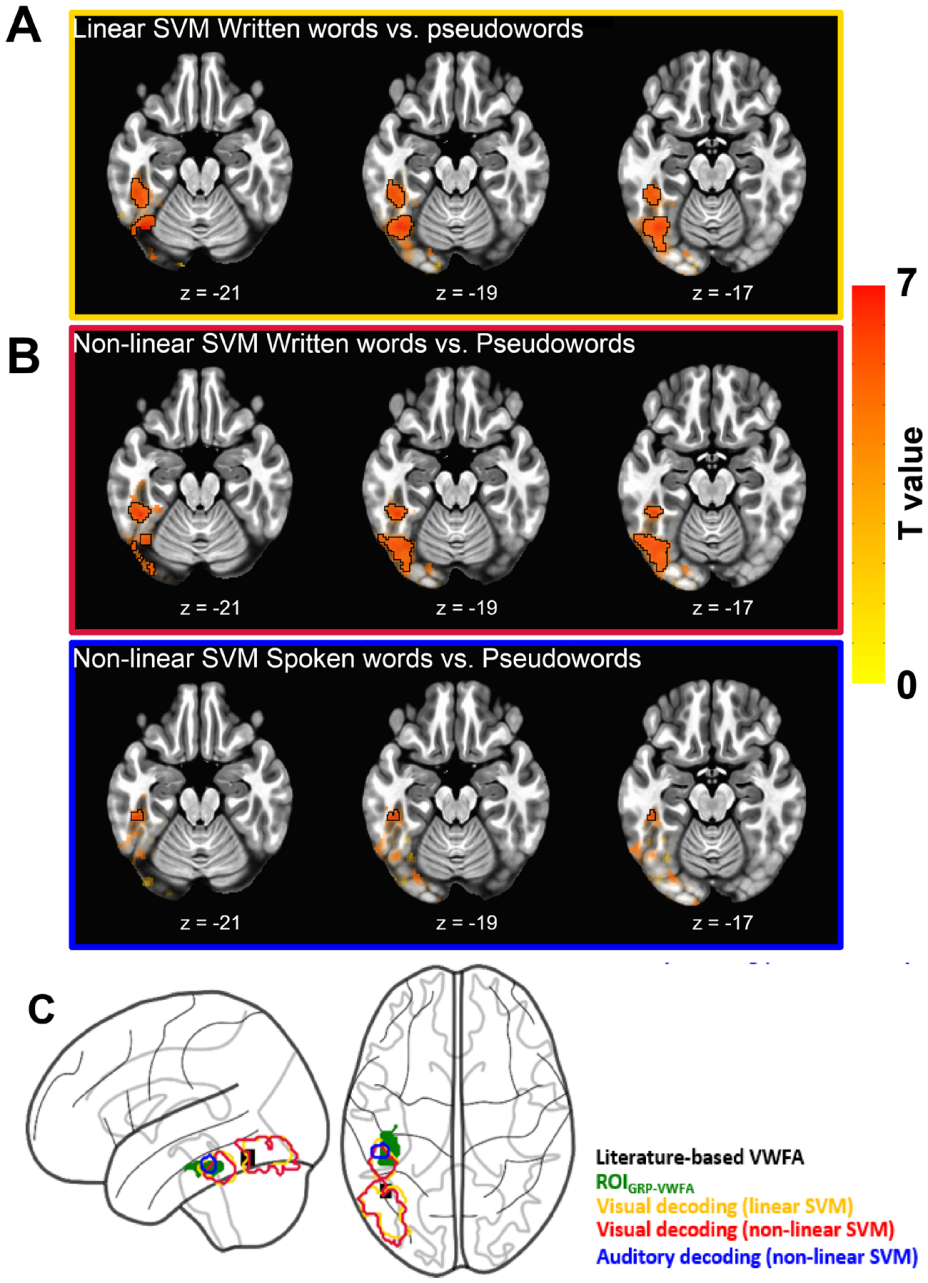
Results of searchlight MVPA for lexicality decoding in the VWFA. (A) Linear SVM revealed two significant clusters with above-chance-level accuracies for written inputs (FWE*p*< 0.05, voxel-wise*p*< 0.005; highlighted by black contours). (B) Non-linear SVM revealed two significant clusters for written inputs and one significant cluster for spoken inputs (FWE*p*< 0.05, voxel-wise*p*< 0.005; highlighted by black contours). Sub-threshold results were shown outside black contours and visible with decreasing opacity as the corresponding statistical significance decreases. (C) Glass brain showing the overlap between the ROI_GRP-VWFA_(green patch) and the clusters that showed above-chance visual (yellow and red contour for the linear and non-linear SVM, respectively) and auditory decoding performance (blue contour). The black patch indicates the literature-based VWFA reported in a meta-analysis by Jobard et al. (2003: MNI -44 ± 4, -58 ± 5, -15 ± 6)

Within the VVP mask (Fig. 4B, orange contour), the searchlight analysis using linear SVM only showed two significant clusters with above-chance-level accuracies for written inputs (FWE*p*< 0.05, voxel-wise*p*< 0.005;Fig. 5A). The first cluster was centered in the posterior fusiform gyrus extending into the inferior occipital cortex (peak MNI -40, -78, -14). The second one was centered in the anterior fusiform gyrus and largely overlapped with the VWFA (peak MNI -46, -41, -22). No significant clusters were found for auditory or cross-modal decoding. Interestingly, the non-linear SVM revealed a significant cluster that had above-chance-level accuracy for spoken inputs, around the VWFA where the within-modal auditory RSE was observed (Fig. 5B,5C; FWE*p*< 0.05, voxel-wise*p*< 0.005; peak MNI -51, -34, -19), as well as two clusters with above-chance-level accuracies for decoding written inputs. These two clusters are at the similar locations as those obtained in the linear SVM (FWE*p*< 0.05, voxel-wise*p*< 0.005; peak MNI -54, -64, -9 and -46, -43, -21;Fig. 5B,5C). Note that neither linear nor non-linear SVM led to an above-chance-level accuracy in the cross-modal conditions^1^. We further applied a simple linear classifier Linear Discriminant Analysis (LDA) and a non-linear Gradient Boosting Classifier (GBC) with searchlight MVPA and confirmed the results obtained above (Supplementary Text 7,Supplementary Fig. S4).

To confirm the validity of the cross-modal MVPAs, the search areas were extended to multimodal language areas (Price, 2012). The same searchlight analysis was conducted using a mask that covered high-order language regions, i.e., left middle and superior temporal gyri, left supramarginal gyrus, and left angular gyrus. The analysis showed a significant decoding performance in all conditions in the left pSTS and left temporoparietal junction (Supplementary Text 8,Supplementary Fig. S5A, S5B), that is, areas reported to be involved in multimodal language processing (Hocking & Price, 2008;Regev et al., 2013;Spitsyna et al., 2006). Note that the left pSTS cluster revealed by the searchlight MVPA overlapped with the multimodal ROI reported in Erickson et al.’s meta-analysis on audiovisual integration (Erickson et al., 2014), which also showed significant RSEs (using univariate analyses) in both within-modal and cross-modal conditions (Supplementary Text 8,Supplementary Figs. S3C and S4C).

### 3.2 Experiment 2: sEEG

#### 3.2.1 Exploratory analyses of the temporal dynamics of the within-modal visual and within-modal auditory repetition suppression effects

The converging results obtained in Experiment 1 suggested that the VWFA responses to written and spoken inputs could reflect the existence of populations of unimodal neurons that are activated by either auditory or visual language modality (Pattamadilok et al., 2019). To complement the above fMRI observations by exploring the temporal dynamics of the VWFA response to spoken and written inputs, we analyzed high-frequency activity (70-150 Hz) in sEEG recordings.

**Fig. 6.**
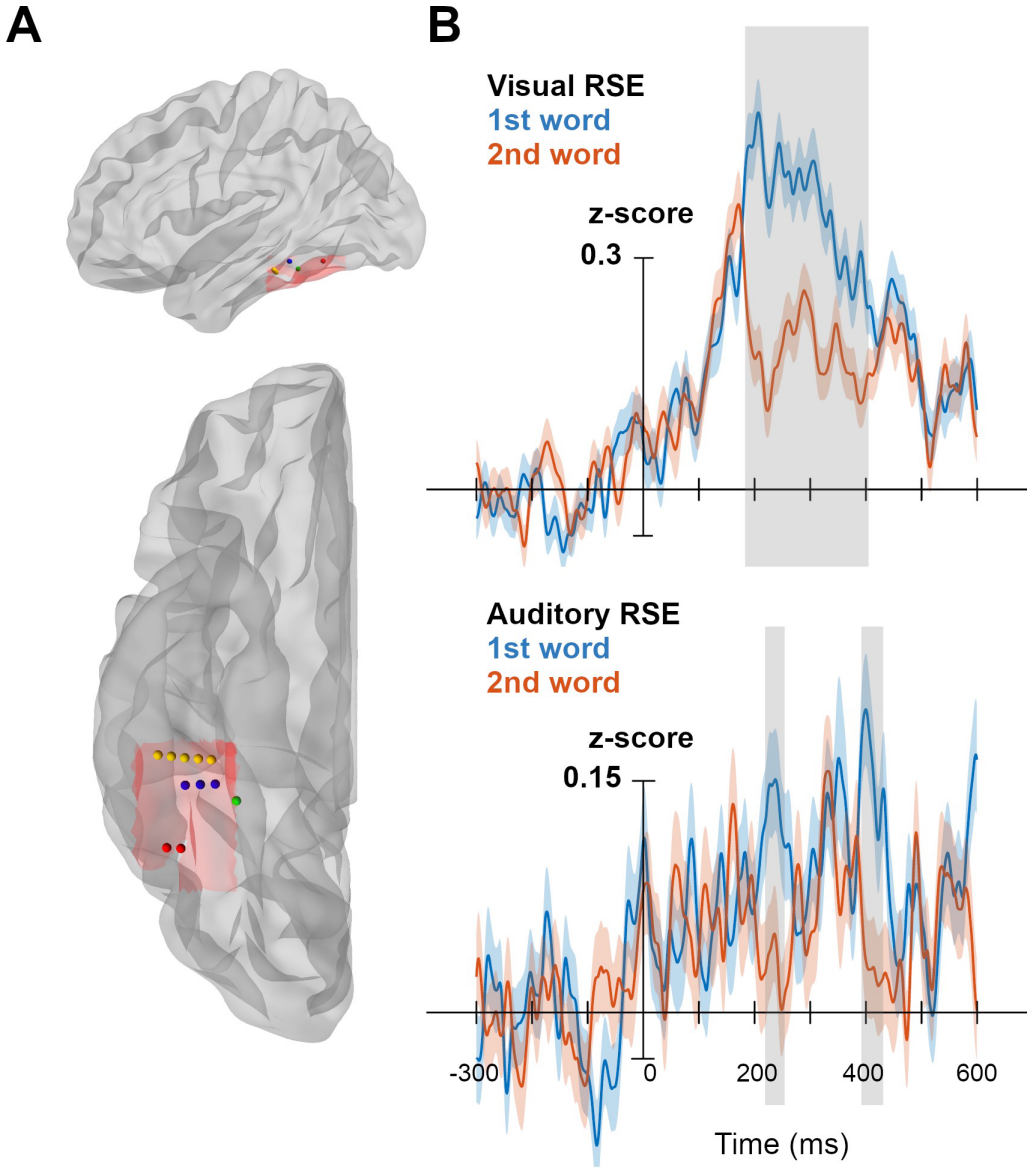
Temporal dynamics of the within-modal visual and within-modal auditory RSEs. (A) The locations of the 11 electrodes in four patients (illustrated in four colors). The red shadow on the cortex view indicates the box mask used for electrode selection. (B) Time courses of high-frequency activity (HFA) averaged across 11 electrodes recorded on the 1^st^and the 2^nd^word of the same-word pairs presented in the*SameVV*(upper panel) and*SameAA*(lower panel) conditions. The gray bands represent time-windows where the within-modal visual (upper panel) and the within-modal auditory (lower panel) RSEs are significant, i.e., higher brain signal on the 1^st^than on the 2^nd^word within a pair [*p*< 0.05, permutation tests with multiple comparisons correction (Dubarry et al., 2022)]. Ribbons represent standard errors across trials.

The electrode localization and selection criteria resulted in 11 contacts across the four patients (Fig. 6A). The analysis of the 11 contacts showed significant within-modal visual and within-modal auditory RSEs [*p*< 0.05, permutation tests with multiple comparisons correction (Dubarry et al., 2022)]. The significant within-modal visual RSE was observed in a time-window spanning from 183 ms to 405 ms (duration = 222 ms) and peaked at 206 ms (Fig. 6Btop panel). The significant within-modal auditory RSE was observed at two distinct temporal stages, that is, from 219 ms to 254 ms (duration = 35 ms) with a peak at 246 ms, which overlapped with the initial phase of the within-modal visual RSE and from 392 ms to 431 ms (duration = 39 ms) with a peak at 401 ms (Fig. 6Bbottom panel), which immediately followed the time-window of the within-modal visual RSE. Regarding the RSEs in the cross-modal conditions, an exploration of the brain responses showed a strong response bias to visual input. As illustrated inSupplementary Fig. S6, the present VWFA electrodes showed higher activity to visual words compared to auditory words regardless of their position in the word pair. This strong response to visual input masked any potential cross-modal RSE. Note, however, that such bias could not affect the computation of the cross-modal RSEs in the fMRI experiment since the RSEs were computed from the global brain activity of the two words within a pair rather than from each individual word. Thus, even though such bias had been present, it would have been cancelled out in the*same-word pair*vs.*different-word pair*contrast. Altogether, the results obtained in the sEEG study mainly informed us about the temporal dynamics of the VWFA’s unimodal responses to the visual and auditory input without providing conclusive information on the existence of cross-modal RSEs.

## 4 Discussion

Learning to associate visual symbols with speech sounds is the fundamental stage of reading acquisition. Considering the role of the VWFA in this learning process and its location at the interface between the visual cortex and the temporal language areas, it is not surprising that this area also responds to speech, in addition to its consistent responses to known scripts. The present study examined the underlying mechanism(s) of the VWFA responses to spoken inputs through fine-grained analyses of both fMRI and sEEG data, aiming to reveal the nature of the representations encoded by neural populations within the VWFA and the temporal dynamics of the process. The analyses presented here provided converging evidence in favor of the*Heterogeneous Neural Populations hypothesis*(Price & Devlin, 2003,^2011^).

Mainly, the repetition suppression protocol in both fMRI and sEEG experiments revealed significant within-modal visual and auditory RSEs, with no reliable evidence for cross-modal RSEs. These two within-modal RSEs respectively suggest the existence of written language coding and spoken language coding neural populations in the VWFA. In terms of spatial distribution, the winner-take-all analysis conducted on the fMRI data showed that these two types of neural populations intermingled within the area, with a strong inter-individual variability in the distribution of the voxels that showed a preference for either within-modal visual RSE or within-modal auditory RSE. Interestingly, this heterogeneous neural populations pattern appears to be specific to the VWFA, as the other six ROIs along the ventral visual pathway only exhibited significant within-modal visual RSEs associated with a trend for an enhancement of within-modal auditory RSE from the posterior to anterior parts of the pathway. This trend is in line withTaylor et al.’s finding (2019)that reported a posterior-to-anterior gradient of neural representations reflecting information about phonological form, with the highest level of sensitivity to phonology being observed in the VWFA. It is worth noting that the specific location of the VWFA varies across studies. In the present study, the region that we defined as VWFA was functionally localized by a visual localizer task rather than being extracted from the literature. The location of this area where we observed heterogeneous neural populations through RSEs and MVPA decoding was more anterior than the VWFA identified byCohen et al.(2000),Dehaene et al. (2010), and a meta-analysis conducted by Jobard et al. (2003, seeFig. 5Cand S4C for illustrations), while it was similar to the locations reported by Swzed et al. (2014) andZhan et al. (2023). As discussed inZhan et al. (2023), a more anterior location of VWFA could to some extent be due to the optimization of MRI acquisition. Indeed, we applied a partial coverage sequence to increase the imaging resolution and the signal-to-noise ratio in our study.

Regarding the temporal dynamics of the RSEs, the sEEG data revealed a significant within-modal visual RSE over a large time-window and a significant within-modal auditory RSE at two distinct stages that coincided with the onset and the offset of the within-modal visual RSE. Although this exploratory result calls for a larger scale study, they suggested that the presence of the within-modal auditory RSE in two separated stages might be supported by two distinct mechanisms. First, the early within-modal auditory RSE that overlapped with the onset of the within-modal visual RSE suggests that the spoken language coding neural populations in the VWFA may directly receive information from the auditory cortex in the same way as the written language coding neural populations that receive information from the early visual regions. Second, the late within-modal auditory RSE may result from the top-down activation of the written language coding neural populations once the phonological input had been converted into orthographic representations. This second mechanism, which is more time consuming, aligns with the*Orthographic Tuning hypothesis*(Dehaene & Cohen, 2011;Dehaene et al., 2010) arguing that the phonological information must be converted into orthographic representations before being processed in the VWFA.

Nevertheless, the above conclusion also relies on the absence of statistically significant cross-modal RSEs, whose origin remains controversial. As argued inSawamura et al.’s study (2006)in which a single-cell recording was conducted in IT neurons in macaque, “*…for the large majority of the neurons that respond similarly to A and B, the degree of adaptation is smaller for the successive presentation of two different stimuli than for a repetition of a stimulus, even when the two different stimuli (i.e., B and A) elicit, on average, a nearly identical response to their first presentation in a sequence*.” (p. 309). Thus, in the present case, it remains possible that, even in neural populations that process both information modalities, the neural adaptation to within-modal repetition, that is, where the repeated stimuli are perceptually identical, is still larger than the neural adaptation to cross-modal repetition due to the repetition of the exact same stimulus in the former condition. A fairer comparison between within-modal and cross-modal RSE could be made by using, in the within-modal conditions, the same written word presented in different fonts or cases and by using the same spoken word pronounced by different speakers. Nevertheless, despite this methodological issue, we provided pieces of evidence suggesting that the absence of cross-modal RSEs within the VWFA could not merely be attributed to this factor. First, we observed a significant cross-modal RSE in the left pSTS, which is considered as a multimodal region in language processing (Erickson et al., 2014;Price, 2012;Supplementary Text 6). Second, the MVPA result led to the same conclusion as the repetition suppression protocol, that is, significant within-modal visual and within-modal auditory decoding performances, but non-significant cross-modal decoding performance in the VWFA. Finally, MVPA also showed a significant cross-modal decoding in the TPJ and pSTS (Supplementary Text 8), which indicated that the analysis remained sufficiently powerful to uncover the cross-modal decoding.

From a methodological point of view, repetition suppression and MVPA are two complementary methods (Barron et al., 2016;Davis & Poldrack, 2013). While repetition suppression relies on temporally adjunct stimuli and measures neural activity at the level of local neural populations (Barron et al., 2016;Grill-Spector et al., 2006;Glezer et al., 2009), MVPA reveals neural representations based on a spatially extended cluster of voxels and, therefore, reflects collective neural responses across voxels within the cluster (Hatfield et al., 2016;Norman et al., 2006;Oosterhof et al., 2013). One intriguing observation regarding the MVPA results that deserves further discussion is the difference between linear and non-linear classifiers: When applying MVPA with a linear classifier, only clusters showing successful visual decoding were detected, whereas the non-linear classifier allowed us to uncover clusters overlapping with VWFA for both visual and auditory decoding. Notably, the visual decoding clusters identified by the linear and non-linear SVM were highly overlapped, thus suggesting that the non-linear classifier not only revealed the same neural representations as did the linear classifier, but also those that the linear classifier failed to detect. The advantage of non-linear over linear classifiers could be due to their flexible decision boundary (Kragel et al., 2012;Rasmussen et al., 2011). In contrast to the predominant written language coding neural populations in the VWFA, the spoken language coding neural populations could be more sparsely distributed (Fig. 3B). Therefore, a flexible decision boundary offered by the non-linear classifier (e.g., polynomial, sinusoid) might show some additional benefits in capturing such complex combinations of activity across voxels.

Overall, we provided converging findings from univariate activation, fMRI/sEEG repetition suppression and non-linear MVPA that support the co-existence of written and spoken language coding neural populations within the VWFA. These observations are in line with the previous observation from a TMS adaptation paradigm (Pattamadilok et al., 2019). Under the multisensory convergence framework proposed byMeredith (2002), the co-existence of the two unimodal neural populations within the VWFA reflects an “areal convergence”. In other words, written and spoken inputs may not converge to the same (multimodal) neurons, but to the same area where the two unimodal neural populations intermingled. Nevertheless, at this stage of research, one could not rule out the existence of multimodal neural populations that respond to both language modalities (i.e., neural convergence, according toMeredith, 2002). In a recent study,Woolnough and Tandon (2024)applied extra-operative or intraoperative stimulation on epileptic patients and reported evidence in favor of multimodal neural populations, where the stimulation disrupted both reading and naming tasks. These neural populations were located between the anteromedial part of the vOT that showed a higher probability of producing naming disruption during the stimulation and the posterolateral part that showed greater reading-specific disruption. While using a different technique, the authors provided evidence that is in line with our conclusion on the existence of heterogeneous populations of neurons within this functional region, although their observation included unimodal visual, unimodal auditory, and multimodal neural populations.

The converging findings obtained across brain imaging techniques (TMS, fMRI, sEEG) open further discussions both on the origin of the VWFA and on the consequence of reading acquisition on functional reorganization of the human brain. Prior research suggests that the emergence of reading specialization in this specific part of the ventral visual pathway is triggered by the mapping between the orthographic and phonological codes during reading acquisition (Brem et al., 2010;Pleisch et al., 2019;Wang et al., 2018). Yet, it remains to be investigated whether this repeated connection between the two language codes has rendered some “visual neurons” sensitive to speech inputs. In such a case, the existence of the spoken language coding neural populations in the VWFA would be a*consequence*of functional reorganization of the visual pathway following reading acquisition (Dehaene et al., 2015;Siuda-Krzywicka et al., 2016). An alternative possibility is that these spoken language coding neurons may*predate*reading acquisition. In the other words, some neurons that are located at the transition between the visual and the spoken language system, and that become the VWFA later on, might already show some degree of sensitivity to spoken language before reading acquisition. This assumption seems plausible given existing findings on brain connectivity. Indeed, a longitudinal study reported that the anatomical connectivity between the “future VWFA” and the temporal language areas predates reading acquisition, and the anatomical connectivity pattern of this specific part of the left ventral visual pathway with the rest of the brain can predict the precise location of the area that will become the VWFA once reading is acquired (Saygin et al., 2016). Moreover, the area already shows functional connectivity with spoken language regions in neonates as early as one week after birth (Li et al., 2020). Given that cross-modal projections can trigger responses to new sensory modalities even in a unimodal area (Allman et al., 2008;Ghazanfar & Schroeder, 2006), the connections between the left ventral visual pathway and spoken language regions could potentially lead to the development of spoken language coding neural populations in the visual system independently of reading acquisition. In either way, these neural populations could facilitate the acquisition of orthographic-phonological mapping when one learns to read. This potential benefit is obvious at the initial stage of reading acquisition, as numerous studies have shown a positive relationship between reading skill in children and the activation of the VWFA during spoken language processing (Desroches et al., 2010;Raschle et al., 2012;Wang et al., 2018,^2021^).

Finally, the co-existence of written and spoken language coding neural populations also offers a new perspective for understanding the connections between the VWFA and other brain regions (Chen et al., 2019;Koyama et al., 2010;Stevens et al., 2017;Wang et al., 2022). It is possible that different neural populations in the area also show different patterns of connectivity, for example, some may establish a direct communication with the spoken language system while others may be more tightly connected with regions in the visual cortex. This perspective suggests a potential benefit of considering the type of neural population in fine-grained connectivity analyses.

Limitations: The present study provides evidence that supports the*Heterogeneous Neural Populations hypothesis*, in line with recent observations reported by other research groups (Van Audenhaege et al., 2024;Woolnough & Tandon, 2024). However, the study also has limitations that call for further investigation. The first limitation is the small sample sizes that might have reduced the statistical power and allowed us to reveal only the most robust effects, that is, the presence of unimodal visual RSE and visual decoding; and the unimodal auditory RSE and auditory decoding. Therefore, the absence of evidence for cross-modal RSEs and decoding should be considered with caution since they could be observed in a larger sample size and/or through more fine-grained techniques such as neuropixels, micro-macro depth electrodes, or single cell recording (Quiroga et al., 2005). These fine-grained techniques could provide insights into what happens at the level of neurons. Another limitation concerns the choice of protocol. Our aim was to investigate the nature of representations encoded in the VWFA by using multiple data modalities and analysis approaches, including BOLD activation, univariate RSEs, multivariate decoding and sEEG activity. Although the different protocols and analyses provided convergent evidence, the combination of multiple approaches also raised an issue about the comparability of their outcomes. For instance, while a univariate analysis was applied to the repetition suppression protocol, a multivariate analysis was used in the lexical decision task. Given that the design of the repetition suppression protocol did not allow us to conduct the MVPA due to a small number of trials, the differences in analysis methods created a gap that made result comparisons less than optimal. In order to confirm our conclusion, several adaptations should be considered in future studies, for instance, by applying the same type of analyses (univariate/multivariate) across tasks, by improving the compatibility of the within-modal and cross-modal conditions. Further evidence could also be obtained from representational similarity analysis looking for similarity between items that share letters and/or sounds within vs. cross-modality. Finally, the role of semantic representations was not examined in the present study. The absence of cross-modal RSE and MVPA decoding makes the interpretation that amodal semantic representations might play a central role in our study unlikely. However, future studies explicitly focusing on the dissociation between multimodal and amodal neural populations in the VWFA can provide further insights into the content of information processed in this brain area.

Based on the present findings, the VWFA exhibited significant activation, repetition suppression effects and successful decoding performance to both written and spoken inputs. Our study provided converging evidence for the co-existence of at least written and spoken language coding neural populations in the VWFA, in line with the*Heterogeneous Neural Populations hypothesis*(Price & Devlin, 2003,^2011^). These observations not only provide insight into the nature of the representations encoded in one of the most important areas of the reading network, but also open further discussions on how the human brain may be prepared and adapt for an acquisition of a complex ability such as reading.

## Supplementary Material

Supplementary Material

## Data Availability

The ethical approval does not allow us to share fMRI data publicly in a data repository. The fMRI data are available on Zenodo with restrictions (https://zenodo.org/doi/10.5281/zenodo.12703902). Please send an email to the corresponding author to request access to restricted files. The ethical approval does not allow us to share SEEG data on epileptic patients. All the code necessary for reproducing the fMRI and sEEG results are publicly available at Github (https://github.com/ws1011001/VWFA_multimodal_neural_representations.git).

## Footnotes

1 Note that the failure to observe above-chance-level accuracies for decoding in the cross-modal conditions allowed us to rule out the possibility that the successful decoding reported in the within-modal conditions was merely driven by the behavioral responses that were aligned with the MVPA classifications (words vs. pseudowords).
