# Supplementary material for: Revealing the co-existence of written and spoken language coding neural populations in the visual word form area
Source: Imaging Neurosci (Camb). 2025 Mar 31;3:imag_a_00524. doi: 10.1162/imag_a_00524 (PMC12319854; doi:10.1162/imag_a_00524)
Supplement: Supplementary Material [file imag_a_00524-supp.pdf]

Supplementary Material

Revealing the co-existence of written and spoken language coding neural populations in the visual word form area

Shuai Wang[1,2], Anne-Sophie Dubarry[1,3], Valérie Chanoine[1,2], Julien Sein[4], Jean-Luc Anton[4], Bruno Nazarian[4], Manuel R. Mercier[5,6], Agnès Trébuchon[5,6], Chotiga Pattamadilok[1*]

Supporting Information

Table S1. Statistical properties of the stimuli in Experiment 1 and Experiment 2

Table 2S: Summary of p. values, Cohen's d and 95% Confidence Interval (CI) for ROI-based comparisons obtained in the auditory task and the repetition suppression protocol

Text 1. fMRI Data Pre-processing.

Text 2. Multi-patient permutation tests (sEEG).

Text 3. Identification of individual ROI ($ROI_{IND-VWFA}$) within the left-vOT.

Text 4. Examination of individual ROIs responses to spoken inputs and its repetition suppression pattern.

Text 5. Voxel-wise univariate analysis conducted in the auditory task.

Text 6. Validation of the repetition suppression protocol.

Text 7. MVPA results using Linear Discriminant Analysis (LDA) and Gradient Boosting Classifier (GBC).

Text 8. Multimodal regions revealed by MVPA decoding of stimulus lexicality.

Table S1. Statistical properties of the stimuli in Experiment 1 and Experiment 2 (Mean ± Standard Deviation). Database: http://www.lexique.org/

Task	Condition	Example	Number of letters	Number of syllables	Number of phonemes	Lexical frequency (written)	Lexical frequency (spoken)	OLD20	PLD20	Uniqueness point
Visual localizer task	Words	poste /pɔst/	5.47±0.50	1.59±0.49	4.05±0.76	107.51±27.20	137.15±17.00	1.69±0.27	1.42±0.31	3.96±0.72
	Consonant strings	pclbt	5.47±0.50	-	-	-	-	-	-	-
Auditory task	Words	frère /fʁɛʁ/	5.50±0.50	1.54±0.50	4.14±0.78	58.73 83.36	49.04 80.07	1.70±0.25	1.43±0.34	4.05±0.75
	Pseudowords	sirèle /siʁɛl/	-	1.54±0.50	4.14±0.78	-	-	-	-	-
Repetition suppression protocol (fMRI)	SameVV	stylo /stilo/	5.21±0.93	1.50±0.51	3.88±1.15	42.56±42.99	34.50±47.08	1.68±0.36	1.44±0.36	3.79±1.18
	DiffVV	office / ɔfis/	5.24±0.83	1.50±0.50	3.96±0.94	42.55±51.50	34.51±43.60	1.67±0.30	1.41±0.37	3.85±0.83
	SameAA	visite /vizit/	5.21±0.93	1.50±0.51	3.92±1.10	42.58±46.48	34.57±37.97	1.69±0.28	1.40±0.33	3.88±1.12
	DiffAA	asile /azil/	5.17±0.92	1.50±0.50	3.97±0.90	42.57±71.52	34.53±51.53	1.65±0.35	1.42±0.37	3.83±0.89
	SameVA	loisir /lwaziR/	5.25±0.90	1.50±0.51	3.92±0.78	42.52±57.66	34.58±41.61	1.63±0.30	1.44±0.32	3.83±0.82
	DiffVA	crèche /kʁɛʃ/	5.28±0.70	1.50±0.50	3.93±0.79	42.54±70.51	34.53±73.63	1.67±0.30	1.39±0.33	3.89±0.78
	SameAV	trafic /tRafik/	5.25±0.85	1.50±0.51	3.92±0.97	42.55±56.78	34.48±69.33	1.70±0.25	1.41±0.35	3.88±0.95
	DiffAV	règle /ʁɛgl/	5.25±0.73	1.50±0.50	3.97±0.86	42.60±49.60	34.57±46.57	1.64±0.30	1.38±0.35	3.86±0.81
	p. value >		*0.99*	*0.99*	*0.99*	*0.49*	*0.55*	*0.95*	*0.98*	*0.99*
Lexical decision task	Words	cadeau /kado/	5.17±0.85	1.47±0.50	3.87±0.98	55.50±73.46	36.73±47.64	1.64±0.34	1.37±0.37	3.77±0.93
	Pseudowords	chantu /ʃɑ̃te/	5.15±1.29	1.45±0.50	3.92±0.93	-	-	-	-	-
	p. value >		*0.8*	*0.9*	*0.99*	-	-	-	-	-
Repetition suppression protocol (sEEG)	SameV1V2	chèque /ʃɛk/	5.08±0.85	1.36±0.48	3.70±0.74	59.26±110.52	54.74±117.57	1.58±0.27	1.28±0.35	3.68±0.71
	SameA1A2	papier /papje/	4.90±0.84	1.30±0.46	3.44±0.84	51.81±86.17	47.84±93.13	1.56±0.30	1.21±0.26	3.42±0.86
	SameV1A2	pignon /piɲɔ̃/	4.92±0.97	1.30±0.46	3.42±0.86	51.83±59.57	47.82±83.87	1.58±0.30	1.25±0.34	3.42±0.86

SameA1V2	herbe /ɛʁb/	4.88±0.80	1.36±0.48	3.44±0.81	51.83±74.14	47.83±64.89	1.58±0.32	1.24±0.30	3.42±0.81
p. value >		*0.58*	*0.84*	*0.4*	*0.66*	*0.94*	*0.98*	*0.98*	*0.4*

Table S2: Summary of p. values, Cohen's d and Confidence Interval (CI) 95% for ROI-based comparisons obtained in the auditory task and the repetition suppression protocol

ROIs	Task and contrast					
	Auditory task: Spoken pseudowords - Scrambled	Auditory task: Spoken words - Scrambled	Repetition suppression protocol: DiffVV - SameVV	Repetition suppression protocol: DiffAA - SameAA	Repetition suppression protocol: DiffVA - SameVA	Repetition suppression protocol: DiffAV - SameAV
ROIvwfa	p < 0.003 Cohen's d: 1.29 (95% CI: 0.62, 1.95)	p < 0.002 Cohen's d: 1.31 (95% CI: 0.64, 1.98)	p < 0.0033 Cohen's d: 0.85 (95% CI: 0.21, 1.48)	p < 0.042 Cohen's d: 0.33 (95% CI -0.28, 0.94)	p > 0.079 Cohen's d: 0.34 (95% CI: -0.27, 0.95)	p > 0.065 Cohen's d: 0.31 (95% CI: -0.31, 0.92)
ROI-40mm	p < 0.011 Cohen's d: 1.00 (95% CI: 0.35, 1.65)	p < 0.008 Cohen's d: 0.99 (95% CI: 0.34, 1.63)	p < 0.012 Cohen's d: 0.44 (95% CI: -0.17, 1.06)	p > 0.097 Cohen's d: 0.26 (95% CI: -0.36, 0.87)	p > 0.10 Cohen's d: 0.35 (95% CI: -0.26, 0.96)	p > 0.46 Cohen's d: 0.01 (95% CI: -0.60, 0.62)
ROI-48mm	p > 0.22 Cohen's d: 0.44 (95% CI: -0.18, 1.05)	p > 0.18 Cohen's d: 0.43 (95% CI: -0.18, 1.05)	p < 0.034 Cohen's d: 0.37 (95% CI: -0.24, 0.99)	p > 0.25 Cohen's d: 0.12 (95% CI: -0.49, 0.72)	p > 0.11 Cohen's d: 0.32 (95% CI: -0.30, 0.93)	p > 0.20 Cohen's d: 0.15 (95% CI: -0.46, 0.76)
ROI-56mm	p > 0.23 Cohen's d: 0.35 (95% CI: -0.26, 0.97)	p > 0.28 Cohen's d: 0.43 (95% CI: -0.19, 1.04)	p < 0.009 Cohen's d: 0.41 (95% CI: -0.21, 1.02)	p > 0.16 Cohen's d: 0.16 (95% CI: -0.45, 0.77)	p > 0.16 Cohen's d: 0.25 (95% CI: -0.36, 0.86)	p > 0.16 Cohen's d: 0.14 (95% CI: -0.47, 0.74)
ROI-64mm	p > 0.26 Cohen's d: 0.53 (95% CI: -0.09, 1.15)	p > 0.12 Cohen's d: 0.28 (95% CI: -0.33, 0.89)	p < 0.004 Cohen's d: 0.43 (95% CI: -0.18, 1.05)	p > 0.38 Cohen's d: 0.05 (95% CI: -0.56, 0.65)	p > 0.38 Cohen's d: 0.13 (95% CI: -0.48, 0.74)	p > 0.12 Cohen's d: 0.21 (95% CI: -0.40, 0.82)
ROI-80mm	p > 0.57 Cohen's d: 0.14 (95% CI: -0.47, 0.75)	p > 0.57 Cohen's d: -0.17 (95% CI: -0.78, 0.44)	p < 0.006 Cohen's d: 0.35 (95% CI: -0.26, 0.97)	p > 0.84 Cohen's d: 0.03 (95% CI: -0.57, 0.64)	p > 0.84 Cohen's d: -0.17 (95% CI: -0.78, 0.44)	p > 0.84 Cohen's d: 0.02 (95% CI: -0.59, 0.63)

ROI-96mm	p > 0.078<br>Cohen's d: -0.44<br>(95% CI: -1.06, 0.17)	p > 0.058<br>Cohen's d: -0.50<br>(95% CI: -1.12, 0.12)	p > 0.44<br>Cohen's d: 0.09<br>(95% CI: -0.52, 0.70)	p > 0.44<br>Cohen's d: 0.03<br>(95% CI: -0.58, 0.64)	p > 0.44<br>Cohen's d: 0.08<br>(95% CI: -0.53, 0.69)	p > 0.29<br>Cohen's d: 0.26<br>(95% CI: -0.35, 0.87)

Text 1. fMRI Data Pre-processing. The T1-weighted image was corrected for intensity non-uniformity with N4BiasFieldCorrection in ANTs (Avants et al., 2008; Tustison et al., 2010), and used as T1w-reference throughout the workflow. The T1w-reference was then skull-stripped. The brain-extracted T1w was used for segmentation of cerebrospinal fluid (CSF), white-matter (WM) and gray-matter (GM) using fast (FSL 5.0.9). Volume-based spatial normalization to the standard MNI space was performed through nonlinear registration with antsRegistration, using brain-extracted versions of both T1w reference and the T1w template (MNI152NLin2009cAsym). For functional images, the fieldmap distortion correction was performed based on a phase-difference map. The functional images were then co-registered to the T1w reference using flirt (FSL 5.0.9) with the boundary-based registration (Greve et al., 2009) with nine degrees of freedom. Head-motion parameters were estimated before any spatiotemporal filtering using mcflirt (FSL 5.0.9). Fieldmap distortion correction, head-motion correction, BOLD-to-T1w co-registration, and spatial normalization were carried out in a single interpolation step by composing all the pertinent transformations. The pre-processed BOLD data were then used to calculate several confounding time series, including framewise displacement (FD), the mean signals within the white matter and the CSF, and a set of principal components of white matter and CSF that were extracted by the aCompCor method (Behzadi et al., 2007).

Text 2. Multi-patient permutation tests (sEEG). For each trial, time-frequency power was computed on consecutive 10 Hz bands between 70 and 150 Hz with a 7 cycles Morlet wavelet. Baseline correction was applied at each 10 Hz band by

calculating a z-score relative to activity during the baseline from 300 ms to 10 ms before trial onset to exclude edge effects. All bands were then averaged and a two-tailed unpaired t-test was performed at each time point. In order to correct for multiple comparisons in the time domain, a permutation approach was conducted. Specifically, for between-condition tests, the trials of two conditions were randomly permuted at each time point for calculating the maximum number of consecutive time points passing the significance threshold (p < .05 unc.). The permutations were conducted 1000 times to obtain a distribution of 1000 maximum numbers. The minimum duration threshold was defined at the right-tail 95% quantile of this distribution.

Text 3. Identification of individual ROI ($ROI_{IND\text{-}VWFA}$) within the left-vOT. In each participant, the peak coordinates and the corresponding T-value were obtained from the first-level (individual) comparison of *words* and *consonant strings*. Participants whose peak activation was significant at p < 0.001 (T > 3.32) were included, resulting in 18 participants with peaks in the ranges of MNI x = [-56, -33], y = [-64, -29], z = [-28, -12] (**Fig. S1A**). The individual ROIs were defined by creating an 8 mm sphere (389 voxels) centered at the peak coordinates. The 8mm radius resulted in a spherical ROI with a similar volume as $ROI_{GRP\text{-}VWFA}$ which contained 311 voxels. As shown in **Fig. S1B**, the individual ROIs ($ROI_{IND\text{-}VWFA}$) had higher activation in *words* than in *consonant strings* (p < 0.0007).

Text 4. Examination of individual ROIs responses to spoken inputs and its repetition suppression pattern. The ROI-based analysis conducted on the $ROI_{IND\text{-}VWFA}$ confirmed the results that we obtained on the $ROI_{GRP\text{-}VWFA}$. As shown in **Fig. S1C**, in the auditory task, $ROI_{IND\text{-}VWFA}$ showed higher activation in response to

spoken words and *spoken pseudowords* than to s*crambled stimuli* (all *p*s < 0.0023 unc.) and no difference between *spoken words* and *spoken pseudowords* was found (*p* > 0.39 unc.). In the repetition suppression protocol, $ROI_{IND\text{-}VWFA}$ showed significant within-modal RSEs in both visual and auditory modalities (*p* < 0.016 unc. and *p* < 0.046 unc., respectively; **Fig. S1D**). As in the main analysis, no cross-modal RSE was observed (all *p*s > 0.09 unc.).

Text 5. Voxel-wise univariate analysis conducted in the auditory task. The whole-brain analysis revealed strong activation in the VWFA for both the *spoken pseudowords - scrambled stimuli* and *spoken words - scrambled stimuli* contrasts (FWE *p* < 0.05, voxel-wise *p* < 0.005; **Fig. S2** axial views), whereas no significant activation was found for the *spoken words - spoken pseudowords* contrast. The bilateral temporal, precentral and postcentral regions also showed the same activation pattern (FWE p < 0.05, voxel-wise *p* < 0.005; **Fig. S2** surface views).

Text 6. Validation of the repetition suppression protocol. In our main analyses, the examination of the RSEs within the VWFA only revealed significant within-modal RSEs (**Fig. 3**). Here, we validated the existence of the pure auditory RSE and cross-modal RSEs by extending the ROI-based analysis to left temporal regions. First, we conducted the analysis on the left and right STG representing the primary auditory cortex. Both STGs were extracted from the AAL template (Rolls et al., 2020) and confined with the group-averaged gray matter mask. As expected, both left and right STG only showed auditory RSE (**Fig. S3A** and **S3B**; *p* < 0.0039 and *p* < 0.0052, respectively; permutation tests with FWE correction for each ROI). Second, we identified a focus in the left pSTS considered as high-level multimodal audiovisual integration area, according to a meta-analysis study (Erickson et al., 2014). This ROI

was defined by creating an 8 mm sphere centered at the focus' coordinates (MNI -54, -44, 8). It also overlapped with the foci involved in the integration of speech and orthographic information reported by van Atteveldt et al. (2004) (TAL -54, -48, 9) and Rueckl et al. (2015) (MNI -48 -42 6). As shown in **Fig. S3C**, this ROI showed significant within-modal and cross-modal RSEs (all *p*s < 0.045; permutation tests with FWE correction).

Text 7. MVPA results using Linear Discriminant Analysis (LDA) and Gradient Boosting Classifier (GBC). To confirm the MVPA results using linear and non-linear SVMs, the searchlight analysis was conducted using another simple linear classifier Linear Discriminant Analysis (LDA) and a non-linear tree-based Gradient Boosting Classifier (GBC), which has a very flexible decision boundary. In line with the results of SVMs, the linear classifier LDA only showed two significant clusters with above-chance-level accuracies for written inputs (FWE $p < 0.05$, voxel-wise $p < 0.005$; **Fig. S4A**). The first cluster was centered in the posterior fusiform gyrus extending into the inferior occipital cortex (peak MNI -40, -76, -12). The second one was centered in the anterior fusiform gyrus and largely overlapped with the VWFA (peak MNI -47, -40, -22). The non-linear GBC revealed a significant cluster that had above-chance-level accuracy for spoken inputs around the VWFA (**Fig. S4B** and **S4C**; FWE $p < 0.05$, voxel-wise $p < 0.005$; peak MNI -39, -20, -22), as well as three clusters with above-chance-level accuracies for decoding written inputs. These three clusters are at the similar locations as those obtained in the linear SVM (FWE $p < 0.05$, voxel-wise $p < 0.005$; peak MNI -49, -64, -7; -49, -43, -26 and -35, -85, -21). Note that neither linear LDA nor non-linear GBC led to an above-chance-level accuracy in the cross-modal conditions. **Fig. S4C** illustrates the location of literature-

based VWFA (Jobard et al., 2003; MNI -44±4, -58±5, -15±6) relative to the $ROI_{GRP-VWFA}$ where unimodal visual and unimodal auditory RSEs were observed, and to the clusters showing successful unimodal visual and unimodal auditory MVPA decoding.

Text 8. Multimodal regions revealed by MVPA decoding of stimulus lexicality. To search for multimodal clusters in the high-order language regions, we created a mask covering the left middle and superior temporal gyri, left supramarginal gyrus and left angular gyrus from the AAL template (Rolls et al., 2020). The mask was then confined by the group-averaged gray matter mask. The searchlight accuracy maps were first estimated for each of the four decoding conditions with FWE $p < 0.05$, voxel-wise $p < 0.005$ (**Fig. S5A**). Then, we identified the areas that showed a significant decoding performance in all conditions by using a conjunction analysis (**Fig. S5B**). The result revealed two clusters centered at the left pSTS (MNI -61, -40, 1) and left temporoparietal junction (MNI -48, -70, 19), which are involved in multimodal language processing (Spitsyna et al., 2006; Hocking et al., 2008; Regev et al., 2013). As shown in **Fig. S5C**, the cluster in the left pSTS revealed by searchlight MVPA overlapped with the ROI defined from the meta-analysis by Erickson et al. (2014) (**Fig. S3C**).

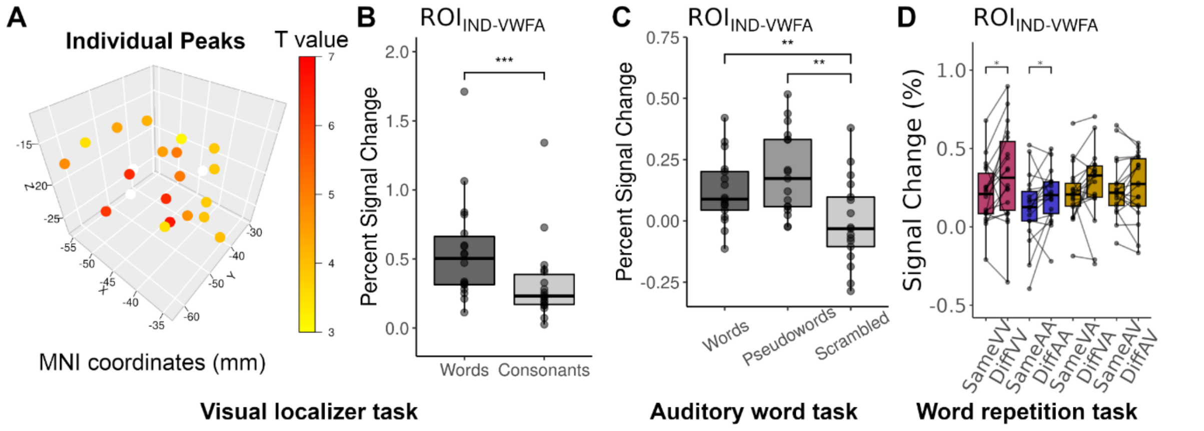

Fig. S1. Identification of individual ROIs corresponding to VWFA ($ROI_{IND\text{-}VWFA}$) and its functional profiles. (A) 3D scatters showing the individual peaks that were significant at $p < 0.001$. In each participant, the individual ROI was created as a sphere centered at the peak coordinates with a radius of 8mm. (B) The $ROI_{IND\text{-}VWFA}$ showed higher activation to *words* compared to *consonant strings*. (C) The $ROI_{IND\text{-}VWFA}$ showed higher activation to *spoken words* and *spoken pseudowords* compared to s*crambled stimuli*. (D) The $ROI_{IND\text{-}VWFA}$ showed significant within-modal visual and within-modal auditory RSEs while no cross-modal RSE was observed.

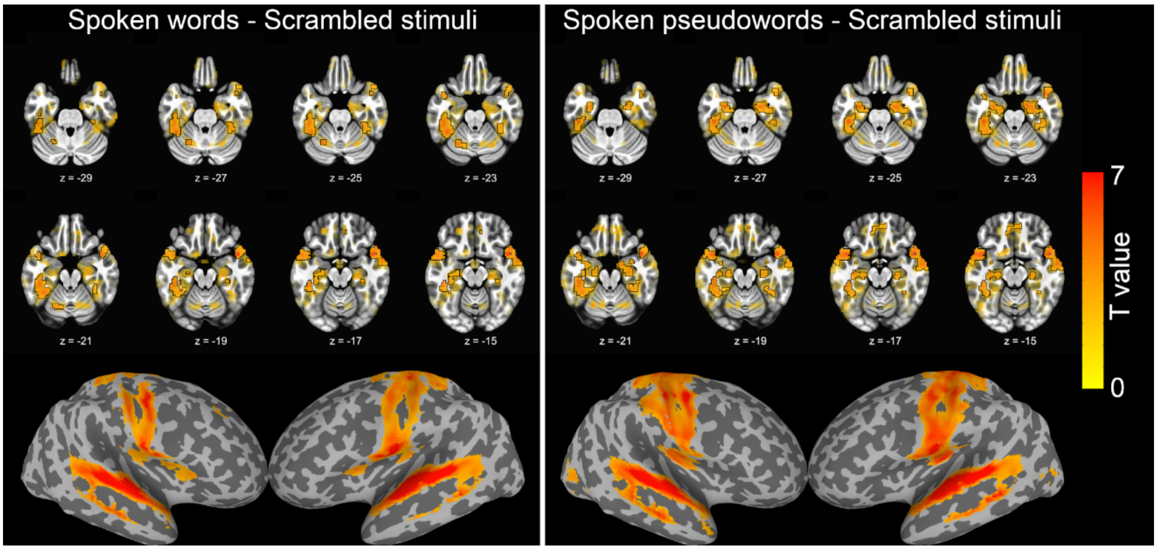

Fig. S2. Activations in the auditory task. Activation maps of brain regions that showed significant activation in the *spoken pseudowords - scrambled stimuli* and *spoken words - scrambled stimuli* contrasts, including left fusiform, bilateral temporal, precentral and postcentral regions (FWE $p < 0.05$, voxel-wise $p < 0.005$; highlighted by black contours). Sub-threshold results were shown outside the black contours and visible with decreasing opacity as the corresponding statistical significance decreases.

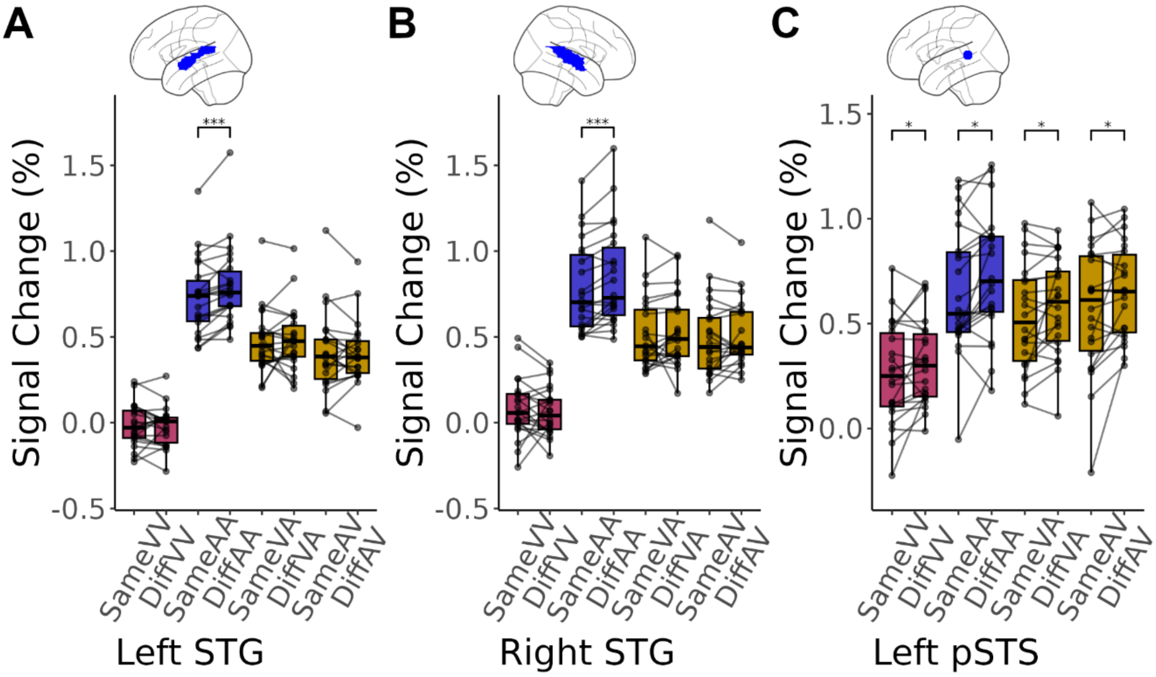

Fig. S3. Repetition suppression effects in primary auditory cortex (bilateral STGs in AAL template) and a multimodal language region in left pSTS. (A) and (B) Both left and right STGs only showed significant within-modal auditory RSE. (C) The ROI in left pSTS showed both within- and cross-modal RSEs. *: $p < 0.05$; **: $p < 0.01$; ***: $p < 0.005$ (permutation tests with FWE correction for each ROI).

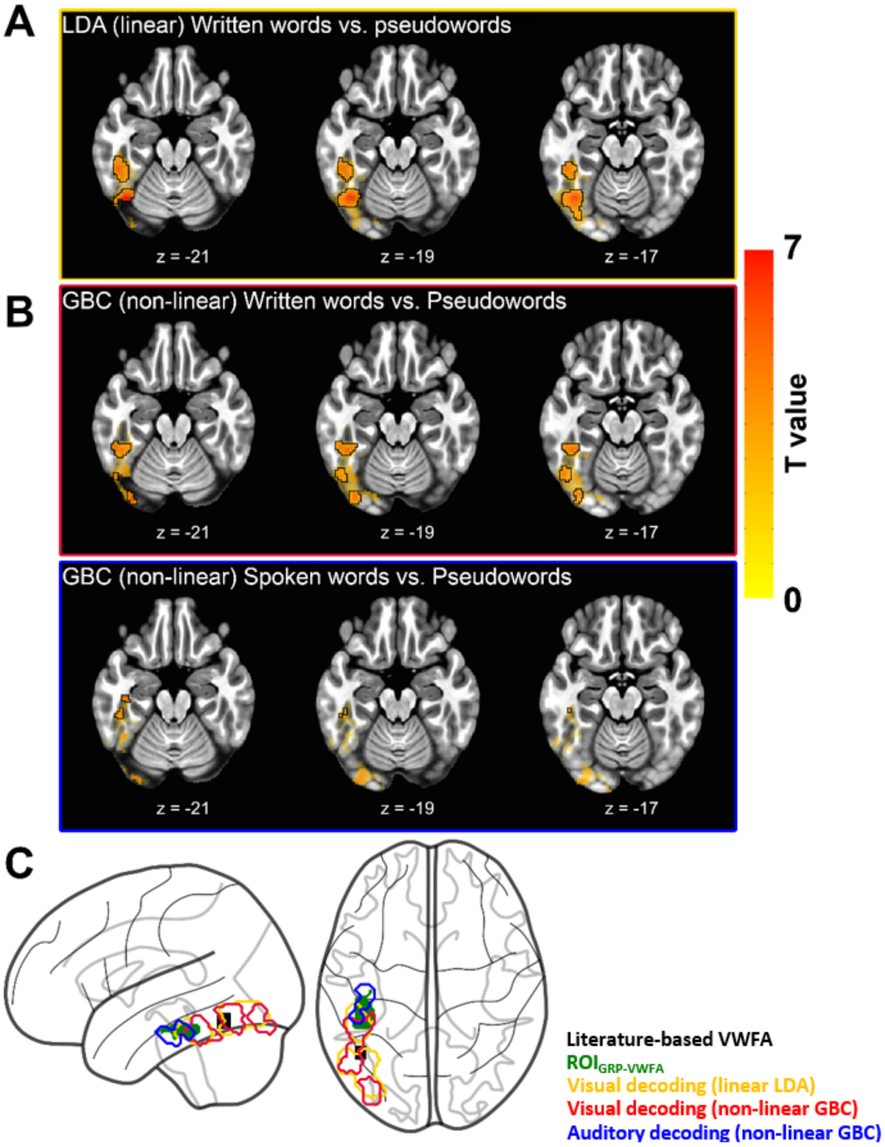

Fig. S4. Results of searchlight MVPA for lexicality decoding in the left ventral visual pathway. (A) The linear classifier LDA revealed two significant clusters that have above-chance-level accuracies for written inputs (FWE $p < 0.05$, voxel-wise $p < 0.005$; highlighted by black contours). (B) The non-linear classifier GBC revealed three significant clusters for written inputs and one significant cluster for spoken inputs (FWE $p < 0.05$, voxel-wise $p < 0.005$; highlighted by black contours). Sub-threshold results were shown outside black contours and visible with decreasing

opacity as the corresponding statistical significance decreases. (C) Glass brain showing the overlap between the $ROI_{GRP\text{-}VWFA}$ (green patch) and the clusters that represented above-chance visual (yellow and red contour for LDA and GBC, respectively) and auditory decoding performance (blue contour). The black patch indicates the literature-based VWFA reported in a meta-analysis by Jobard et al. (2003: MNI -44±4, -58±5, -15±6).

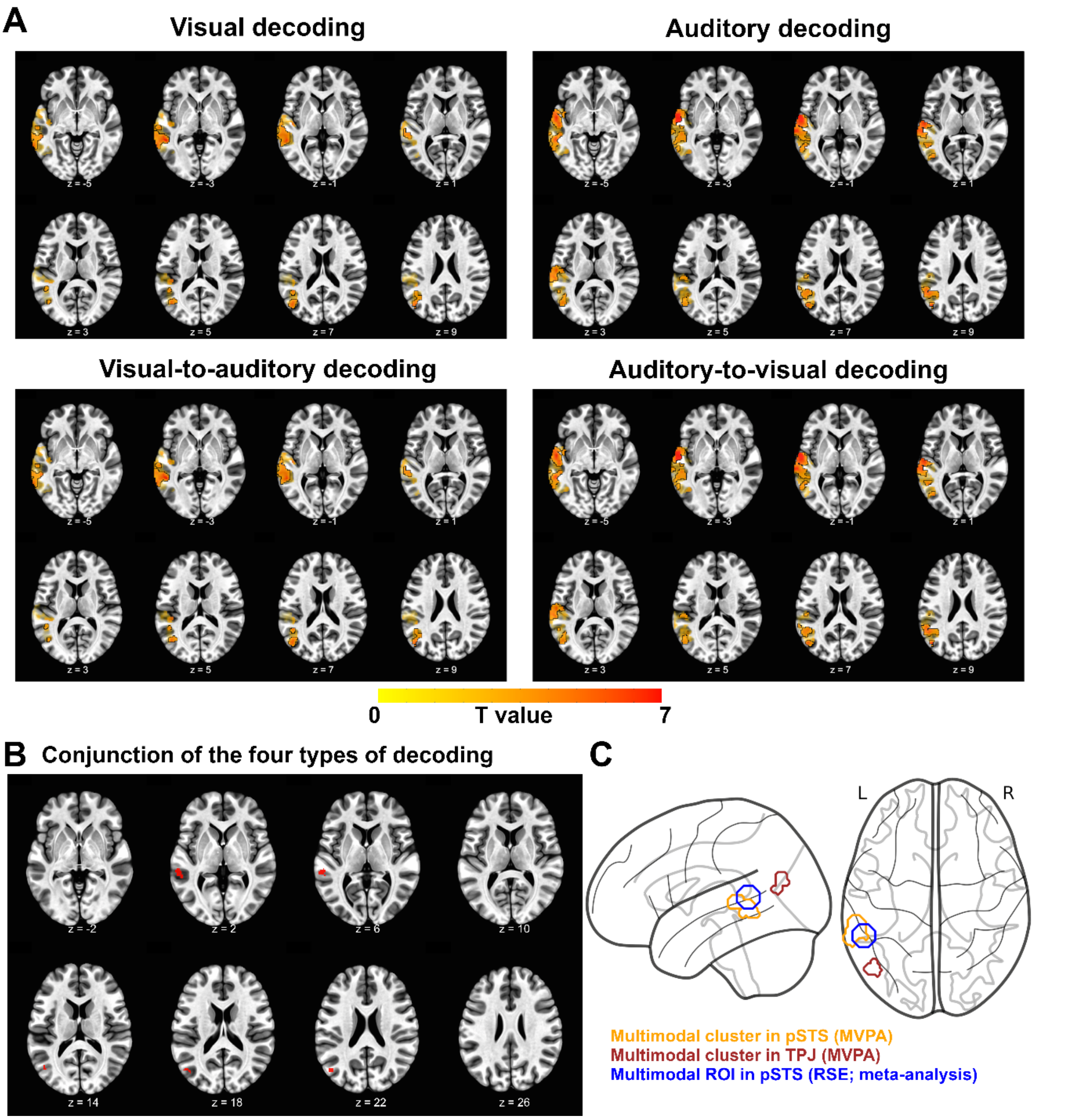

Fig. S5. Searchlight accuracy maps of MVPA decoding of stimulus lexicality in high-order language regions. (A) Brain regions that showed above-chance level accuracy in visual decoding, auditory decoding, visual-to-auditory decoding and auditory-to-visual decoding (FWE $p < 0.05$, voxel-wise $p < 0.005$; highlighted by black contours). Sub-threshold results were shown outside black contours and visible with decreasing opacity as the corresponding statistical significance decreases. (B) Two clusters in the left pSTS and TPJ that showed above-chance

level accuracies in both within- and cross-modal decoding conditions. (C) The multimodal cluster in the left pSTS (orange contour) and the left TPJ (red contour) revealed by MVPA. The former region overlapped with the multimodal ROI that showed both within- and cross-modal RSEs (blue contour).

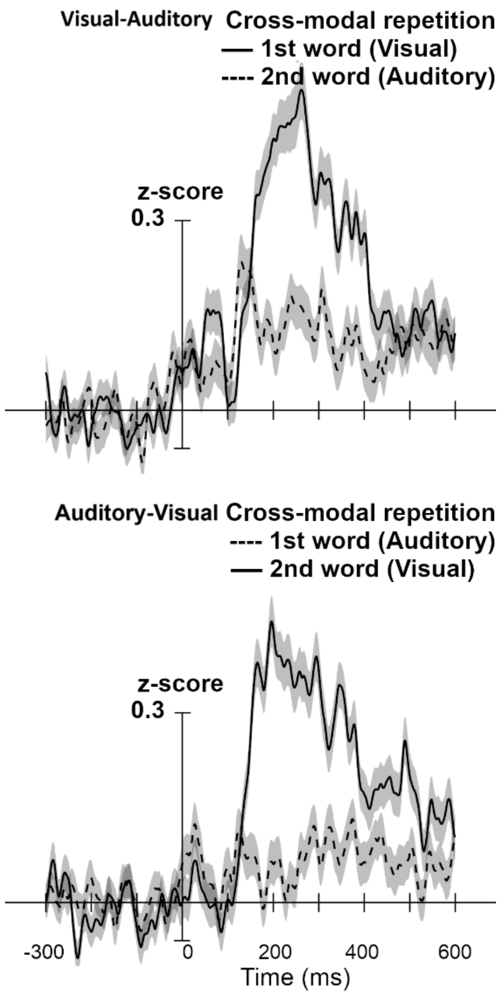

Fig. S6. Temporal dynamics of the cross-modal RSEs. Time courses of high-frequency activity (HFA) averaged across 11 electrodes recorded on the 1st and the 2nd word of the same-word pairs presented in the cross-modal "SameVA" (top panel) and "SameAV" (bottom panel) conditions. Note that the computation of the cross-modal RSEs was biased by the fact that words presented in the visual modality systematically led to higher brain activity regardless of their positions in the word pair.

Supplementary References

Avants, B., Epstein, C., Grossman, M., & Gee, J. (2008). Symmetric diffeomorphic image registration with cross-correlation: Evaluating automated labeling of elderly and neurodegenerative brain. *Medical Image Analysis*, *12*(1), 26–41. https://doi.org/10.1016/j.media.2007.06.004

Behzadi, Y., Restom, K., Liau, J., & Liu, T. T. (2007). A component based noise correction method (CompCor) for BOLD and perfusion based fMRI. *NeuroImage*, *37*(1), 90–101. https://doi.org/10.1016/j.neuroimage.2007.04.042

Erickson, L. C., Heeg, E., Rauschecker, J. P., & Turkeltaub, P. E. (2014). An ALE meta-analysis on the audiovisual integration of speech signals. *Human Brain Mapping*, *35*(11), 5587–5605. https://doi.org/10.1002/hbm.22572

Greve, D. N., & Fischl, B. (2009). Accurate and robust brain image alignment using boundary-based registration. *NeuroImage*, *48*(1), 63–72. https://doi.org/10.1016/j.neuroimage.2009.06.060

Hocking, J., & Price, C. J. (2008). The Role of the Posterior Superior Temporal Sulcus in Audiovisual Processing. *Cerebral Cortex*, *18*(10), 2439–2449. https://doi.org/10.1093/cercor/bhn007

Jobard, G., Crivello, F., & Tzourio-Mazoyer, N. (2003). Evaluation of the dual route theory of reading: A metanalysis of 35 neuroimaging studies. NeuroImage, 20(2), 693–712. https://doi.org/10.1016/S1053-8119(03)00343-4

Regev, M., Honey, C. J., Simony, E., & Hasson, U. (2013). Selective and Invariant Neural Responses to Spoken and Written Narratives. *The Journal of Neuroscience*, *33*(40), 15978–15988.

https://doi.org/10.1523/JNEUROSCI.1580-13.2013

Rolls, E. T., Huang, C.-C., Lin, C.-P., Feng, J., & Joliot, M. (2020). Automated anatomical labelling atlas 3. *NeuroImage*, *206*, 116189. https://doi.org/10.1016/j.neuroimage.2019.116189

Rueckl, J. G., Paz-Alonso, P. M., Molfese, P. J., Kuo, W.-J., Bick, A., Frost, S. J., Hancock, R., Wu, D. H., Mencl, W. E., Duñabeitia, J. A., Lee, J.-R., Oliver, M., Zevin, J. D., Hoeft, F., Carreiras, M., Tzeng, O. J. L., Pugh, K. R., & Frost, R. (2015). Universal brain signature of proficient reading: Evidence from four contrasting languages. *Proceedings of the National Academy of Sciences*, *112*(50), 15510–15515. https://doi.org/10.1073/pnas.1509321112

Spitsyna, G., Warren, J. E., Scott, S. K., Turkheimer, F. E., & Wise, R. J. S. (2006). Converging Language Streams in the Human Temporal Lobe. *The Journal of Neuroscience*, *26*(28), 7328–7336. https://doi.org/10.1523/JNEUROSCI.0559-06.2006

Tustison, N. J., Avants, B. B., Cook, P. A., Yuanjie Zheng, Egan, A., Yushkevich, P. A., & Gee, J. C. (2010). N4ITK: Improved N3 Bias Correction. *IEEE Transactions on Medical Imaging*, *29*(6), 1310–1320. https://doi.org/10.1109/TMI.2010.2046908

Van Atteveldt, N., Formisano, E., Goebel, R., & Blomert, L. (2004). Integration of Letters and Speech Sounds in the Human Brain. *Neuron*, *43*(2), 271–282. https://doi.org/10.1016/j.neuron.2004.06.025
